# Search for massive WH resonances decaying into the $$\ell \nu \mathrm{b} \overline{\mathrm{b}} $$ final state at $$\sqrt{s}=8$$$$~\text {TeV}$$

**DOI:** 10.1140/epjc/s10052-016-4067-z

**Published:** 2016-04-28

**Authors:** V. Khachatryan, A. M. Sirunyan, A. Tumasyan, W. Adam, E. Asilar, T. Bergauer, J. Brandstetter, E. Brondolin, M. Dragicevic, J. Erö, M. Flechl, M. Friedl, R. Frühwirth, V. M. Ghete, C. Hartl, N. Hörmann, J. Hrubec, M. Jeitler, V. Knünz, A. König, M. Krammer, I. Krätschmer, D. Liko, T. Matsushita, I. Mikulec, D. Rabady, B. Rahbaran, H. Rohringer, J. Schieck, R. Schöfbeck, J. Strauss, W. Treberer-Treberspurg, W. Waltenberger, C. -E. Wulz, V. Mossolov, N. Shumeiko, J. Suarez Gonzalez, S. Alderweireldt, T. Cornelis, E. A. De Wolf, X. Janssen, A. Knutsson, J. Lauwers, S. Luyckx, M. Van De Klundert, H. Van Haevermaet, P. Van Mechelen, N. Van Remortel, A. Van Spilbeeck, S. Abu Zeid, F. Blekman, J. D’Hondt, N. Daci, I. De Bruyn, K. Deroover, N. Heracleous, J. Keaveney, S. Lowette, L. Moreels, A. Olbrechts, Q. Python, D. Strom, S. Tavernier, W. Van Doninck, P. Van Mulders, G. P. Van Onsem, I. Van Parijs, P. Barria, H. Brun, C. Caillol, B. Clerbaux, G. De Lentdecker, G. Fasanella, L. Favart, A. Grebenyuk, G. Karapostoli, T. Lenzi, A. Léonard, T. Maerschalk, A. Marinov, L. Perniè, A. Randle-conde, T. Reis, T. Seva, C. Vander Velde, P. Vanlaer, R. Yonamine, F. Zenoni, F. Zhang, K. Beernaert, L. Benucci, A. Cimmino, S. Crucy, D. Dobur, A. Fagot, G. Garcia, M. Gul, J. Mccartin, A. A. Ocampo Rios, D. Poyraz, D. Ryckbosch, S. Salva, M. Sigamani, M. Tytgat, W. Van Driessche, E. Yazgan, N. Zaganidis, S. Basegmez, C. Beluffi, O. Bondu, S. Brochet, G. Bruno, A. Caudron, L. Ceard, G. G. Da Silveira, C. Delaere, D. Favart, L. Forthomme, A. Giammanco, J. Hollar, A. Jafari, P. Jez, M. Komm, V. Lemaitre, A. Mertens, M. Musich, C. Nuttens, L. Perrini, A. Pin, K. Piotrzkowski, A. Popov, L. Quertenmont, M. Selvaggi, M. Vidal Marono, N. Beliy, G. H. Hammad, W. L. Aldá Júnior, F. L. Alves, G. A. Alves, L. Brito, M. Correa Martins Junior, M. Hamer, C. Hensel, C. Mora Herrera, A. Moraes, M. E. Pol, P. Rebello Teles, E. Belchior Batista Das Chagas, W. Carvalho, J. Chinellato, A. Custódio, E. M. Da Costa, D. De Jesus Damiao, C. De Oliveira Martins, S. Fonseca De Souza, L. M. Huertas Guativa, H. Malbouisson, D. Matos Figueiredo, L. Mundim, H. Nogima, W. L. Prado Da Silva, A. Santoro, A. Sznajder, E. J. Tonelli Manganote, A. Vilela Pereira, S. Ahuja, C. A. Bernardes, A. De Souza Santos, S. Dogra, T. R. Fernandez Perez Tomei, E. M. Gregores, P. G. Mercadante, C. S. Moon, S. F. Novaes, Sandra S. Padula, D. Romero Abad, J. C. Ruiz Vargas, A. Aleksandrov, R. Hadjiiska, P. Iaydjiev, M. Rodozov, S. Stoykova, G. Sultanov, M. Vutova, A. Dimitrov, I. Glushkov, L. Litov, B. Pavlov, P. Petkov, M. Ahmad, J. G. Bian, G. M. Chen, H. S. Chen, M. Chen, T. Cheng, R. Du, C. H. Jiang, R. Plestina, F. Romeo, S. M. Shaheen, A. Spiezia, J. Tao, C. Wang, Z. Wang, H. Zhang, C. Asawatangtrakuldee, Y. Ban, Q. Li, S. Liu, Y. Mao, S. J. Qian, D. Wang, M Wang, Z. Xu, C. Avila, A. Cabrera, L. F. Chaparro Sierra, C. Florez, J. P. Gomez, B. Gomez Moreno, J. C. Sanabria, N. Godinovic, D. Lelas, I. Puljak, P. M. Ribeiro Cipriano, Z. Antunovic, M. Kovac, V. Brigljevic, K. Kadija, J. Luetic, S. Micanovic, L. Sudic, A. Attikis, G. Mavromanolakis, J. Mousa, C. Nicolaou, F. Ptochos, P. A. Razis, H. Rykaczewski, M. Bodlak, M. Finger, M. Finger, Y. Assran, S. Elgammal, A. Ellithi Kamel, M. A. Mahmoud, B. Calpas, M. Kadastik, M. Murumaa, M. Raidal, A. Tiko, C. Veelken, P. Eerola, J. Pekkanen, M. Voutilainen, J. Härkönen, V. Karimäki, R. Kinnunen, T. Lampén, K. Lassila-Perini, S. Lehti, T. Lindén, P. Luukka, T. Mäenpää, T. Peltola, E. Tuominen, J. Tuominiemi, E. Tuovinen, L. Wendland, J. Talvitie, T. Tuuva, M. Besancon, F. Couderc, M. Dejardin, D. Denegri, B. Fabbro, J. L. Faure, C. Favaro, F. Ferri, S. Ganjour, A. Givernaud, P. Gras, G. Hamel de Monchenault, P. Jarry, E. Locci, M. Machet, J. Malcles, J. Rander, A. Rosowsky, M. Titov, A. Zghiche, I. Antropov, S. Baffioni, F. Beaudette, P. Busson, L. Cadamuro, E. Chapon, C. Charlot, T. Dahms, O. Davignon, N. Filipovic, A. Florent, R. Granier de Cassagnac, S. Lisniak, L. Mastrolorenzo, P. Miné, I. N. Naranjo, M. Nguyen, C. Ochando, G. Ortona, P. Paganini, P. Pigard, S. Regnard, R. Salerno, J. B. Sauvan, Y. Sirois, T. Strebler, Y. Yilmaz, A. Zabi, J.-L. Agram, J. Andrea, A. Aubin, D. Bloch, J.-M. Brom, M. Buttignol, E. C. Chabert, N. Chanon, C. Collard, E. Conte, X. Coubez, J.-C. Fontaine, D. Gelé, U. Goerlach, C. Goetzmann, A.-C. Le Bihan, J. A. Merlin, K. Skovpen, P. Van Hove, S. Gadrat, S. Beauceron, C. Bernet, G. Boudoul, E. Bouvier, C. A. Carrillo Montoya, R. Chierici, D. Contardo, B. Courbon, P. Depasse, H. El Mamouni, J. Fan, J. Fay, S. Gascon, M. Gouzevitch, B. Ille, F. Lagarde, I. B. Laktineh, M. Lethuillier, L. Mirabito, A. L. Pequegnot, S. Perries, J. D. Ruiz Alvarez, D. Sabes, L. Sgandurra, V. Sordini, M. Vander Donckt, P. Verdier, S. Viret, T. Toriashvili, L. Rurua, C. Autermann, S. Beranek, M. Edelhoff, L. Feld, A. Heister, M. K. Kiesel, K. Klein, M. Lipinski, A. Ostapchuk, M. Preuten, F. Raupach, S. Schael, J. F. Schulte, T. Verlage, H. Weber, B. Wittmer, V. Zhukov, M. Ata, M. Brodski, E. Dietz-Laursonn, D. Duchardt, M. Endres, M. Erdmann, S. Erdweg, T. Esch, R. Fischer, A. Güth, T. Hebbeker, C. Heidemann, K. Hoepfner, S. Knutzen, P. Kreuzer, M. Merschmeyer, A. Meyer, P. Millet, M. Olschewski, K. Padeken, P. Papacz, T. Pook, M. Radziej, H. Reithler, M. Rieger, F. Scheuch, L. Sonnenschein, D. Teyssier, S. Thüer, V. Cherepanov, Y. Erdogan, G. Flügge, H. Geenen, M. Geisler, F. Hoehle, B. Kargoll, T. Kress, Y. Kuessel, A. Künsken, J. Lingemann, A. Nehrkorn, A. Nowack, I. M. Nugent, C. Pistone, O. Pooth, A. Stahl, M. Aldaya Martin, I. Asin, N. Bartosik, O. Behnke, U. Behrens, A. J. Bell, K. Borras, A. Burgmeier, A. Campbell, S. Choudhury, F. Costanza, C. Diez Pardos, G. Dolinska, S. Dooling, T. Dorland, G. Eckerlin, D. Eckstein, T. Eichhorn, G. Flucke, E. Gallo, J. Garay Garcia, A. Geiser, A. Gizhko, P. Gunnellini, J. Hauk, M. Hempel, H. Jung, A. Kalogeropoulos, O. Karacheban, M. Kasemann, P. Katsas, J. Kieseler, C. Kleinwort, I. Korol, W. Lange, J. Leonard, K. Lipka, A. Lobanov, W. Lohmann, R. Mankel, I. Marfin, I.-A. Melzer-Pellmann, A. B. Meyer, G. Mittag, J. Mnich, A. Mussgiller, S. Naumann-Emme, A. Nayak, E. Ntomari, H. Perrey, D. Pitzl, R. Placakyte, A. Raspereza, B. Roland, M. Ö. Sahin, P. Saxena, T. Schoerner-Sadenius, M. Schröder, C. Seitz, S. Spannagel, K. D. Trippkewitz, R. Walsh, C. Wissing, V. Blobel, M. Centis Vignali, A. R. Draeger, J. Erfle, E. Garutti, K. Goebel, D. Gonzalez, M. Görner, J. Haller, M. Hoffmann, R. S. Höing, A. Junkes, R. Klanner, R. Kogler, N. Kovalchuk, T. Lapsien, T. Lenz, I. Marchesini, D. Marconi, M. Meyer, D. Nowatschin, J. Ott, F. Pantaleo, T. Peiffer, A. Perieanu, N. Pietsch, J. Poehlsen, D. Rathjens, C. Sander, C. Scharf, H. Schettler, P. Schleper, E. Schlieckau, A. Schmidt, J. Schwandt, V. Sola, H. Stadie, G. Steinbrück, H. Tholen, D. Troendle, E. Usai, L. Vanelderen, A. Vanhoefer, B. Vormwald, M. Akbiyik, C. Barth, C. Baus, J. Berger, C. Böser, E. Butz, T. Chwalek, F. Colombo, W. De Boer, A. Descroix, A. Dierlamm, S. Fink, F. Frensch, R. Friese, M. Giffels, A. Gilbert, D. Haitz, F. Hartmann, S. M. Heindl, U. Husemann, I. Katkov, A. Kornmayer, P. Lobelle Pardo, B. Maier, H. Mildner, M. U. Mozer, T. Müller, Th. Müller, M. Plagge, G. Quast, K. Rabbertz, S. Röcker, F. Roscher, G. Sieber, H. J. Simonis, F. M. Stober, R. Ulrich, J. Wagner-Kuhr, S. Wayand, M. Weber, T. Weiler, C. Wöhrmann, R. Wolf, G. Anagnostou, G. Daskalakis, T. Geralis, V. A. Giakoumopoulou, A. Kyriakis, D. Loukas, A. Psallidas, I. Topsis-Giotis, A. Agapitos, S. Kesisoglou, A. Panagiotou, N. Saoulidou, E. Tziaferi, I. Evangelou, G. Flouris, C. Foudas, P. Kokkas, N. Loukas, N. Manthos, I. Papadopoulos, E. Paradas, J. Strologas, G. Bencze, C. Hajdu, A. Hazi, P. Hidas, D. Horvath, F. Sikler, V. Veszpremi, G. Vesztergombi, A. J. Zsigmond, N. Beni, S. Czellar, J. Karancsi, J. Molnar, Z. Szillasi, M. Bartók, A. Makovec, P. Raics, Z. L. Trocsanyi, B. Ujvari, P. Mal, K. Mandal, D. K. Sahoo, N. Sahoo, S. K. Swain, S. Bansal, S. B. Beri, V. Bhatnagar, R. Chawla, R. Gupta, U. Bhawandeep, A. K. Kalsi, A. Kaur, M. Kaur, R. Kumar, A. Mehta, M. Mittal, J. B. Singh, G. Walia, Ashok Kumar, A. Bhardwaj, B. C. Choudhary, R. B. Garg, A. Kumar, S. Malhotra, M. Naimuddin, N. Nishu, K. Ranjan, R. Sharma, V. Sharma, S. Bhattacharya, K. Chatterjee, S. Dey, S. Dutta, Sa. Jain, N. Majumdar, A. Modak, K. Mondal, S. Mukherjee, S. Mukhopadhyay, A. Roy, D. Roy, S. Roy Chowdhury, S. Sarkar, M. Sharan, A. Abdulsalam, R. Chudasama, D. Dutta, V. Jha, V. Kumar, A. K. Mohanty, L. M. Pant, P. Shukla, A. Topkar, T. Aziz, S. Banerjee, S. Bhowmik, R. M. Chatterjee, R. K. Dewanjee, S. Dugad, S. Ganguly, S. Ghosh, M. Guchait, A. Gurtu, G. Kole, S. Kumar, B. Mahakud, M. Maity, G. Majumder, K. Mazumdar, S. Mitra, G. B. Mohanty, B. Parida, T. Sarkar, N. Sur, B. Sutar, N. Wickramage, S. Chauhan, S. Dube, K. Kothekar, S. Sharma, H. Bakhshiansohi, H. Behnamian, S. M. Etesami, A. Fahim, R. Goldouzian, M. Khakzad, M. Mohammadi Najafabadi, M. Naseri, S. Paktinat Mehdiabadi, F. Rezaei Hosseinabadi, B. Safarzadeh, M. Zeinali, M. Felcini, M. Grunewald, M. Abbrescia, C. Calabria, C. Caputo, A. Colaleo, D. Creanza, L. Cristella, N. De Filippis, M. De Palma, L. Fiore, G. Iaselli, G. Maggi, G. Miniello, M. Maggi, S. My, S. Nuzzo, A. Pompili, G. Pugliese, R. Radogna, A. Ranieri, G. Selvaggi, L. Silvestris, R. Venditti, P. Verwilligen, G. Abbiendi, C. Battilana, A. C. Benvenuti, D. Bonacorsi, S. Braibant-Giacomelli, L. Brigliadori, R. Campanini, P. Capiluppi, A. Castro, F. R. Cavallo, S. S. Chhibra, G. Codispoti, M. Cuffiani, G. M. Dallavalle, F. Fabbri, A. Fanfani, D. Fasanella, P. Giacomelli, C. Grandi, L. Guiducci, S. Marcellini, G. Masetti, A. Montanari, F. L. Navarria, A. Perrotta, A. M. Rossi, F. Primavera, T. Rovelli, G. P. Siroli, N. Tosi, R. Travaglini, G. Cappello, M. Chiorboli, S. Costa, A. Di Mattia, F. Giordano, R. Potenza, A. Tricomi, C. Tuve, G. Barbagli, V. Ciulli, C. Civinini, R. D’Alessandro, E. Focardi, S. Gonzi, V. Gori, P. Lenzi, M. Meschini, S. Paoletti, G. Sguazzoni, A. Tropiano, L. Viliani, L. Benussi, S. Bianco, F. Fabbri, D. Piccolo, F. Primavera, V. Calvelli, F. Ferro, M. Lo Vetere, M. R. Monge, E. Robutti, S. Tosi, L. Brianza, M. E. Dinardo, S. Fiorendi, S. Gennai, R. Gerosa, A. Ghezzi, P. Govoni, S. Malvezzi, R. A. Manzoni, B. Marzocchi, D. Menasce, L. Moroni, M. Paganoni, D. Pedrini, S. Ragazzi, N. Redaelli, T. Tabarelli de Fatis, S. Buontempo, N. Cavallo, S. Di Guida, M. Esposito, F. Fabozzi, A. O. M. Iorio, G. Lanza, L. Lista, S. Meola, M. Merola, P. Paolucci, C. Sciacca, F. Thyssen, P. Azzi, N. Bacchetta, L. Benato, D. Bisello, A. Boletti, R. Branca, R. Carlin, A. Carvalho Antunes De Oliveira, P. Checchia, M. Dall’Osso, T. Dorigo, U. Dosselli, F. Gasparini, U. Gasparini, A. Gozzelino, K. Kanishchev, S. Lacaprara, M. Margoni, A. T. Meneguzzo, J. Pazzini, N. Pozzobon, P. Ronchese, F. Simonetto, E. Torassa, M. Tosi, M. Zanetti, P. Zotto, A. Zucchetta, G. Zumerle, A. Braghieri, A. Magnani, P. Montagna, S. P. Ratti, V. Re, C. Riccardi, P. Salvini, I. Vai, P. Vitulo, L. Alunni Solestizi, M. Biasini, G. M. Bilei, D. Ciangottini, L. Fanò, P. Lariccia, G. Mantovani, M. Menichelli, A. Saha, A. Santocchia, K. Androsov, P. Azzurri, G. Bagliesi, J. Bernardini, T. Boccali, R. Castaldi, M. A. Ciocci, R. Dell’Orso, S. Donato, G. Fedi, L. Foà, A. Giassi, M. T. Grippo, F. Ligabue, T. Lomtadze, L. Martini, A. Messineo, F. Palla, A. Rizzi, A. Savoy-Navarro, A. T. Serban, P. Spagnolo, R. Tenchini, G. Tonelli, A. Venturi, P. G. Verdini, L. Barone, F. Cavallari, G. D’imperio, D. Del Re, M. Diemoz, S. Gelli, C. Jorda, E. Longo, F. Margaroli, P. Meridiani, G. Organtini, R. Paramatti, F. Preiato, S. Rahatlou, C. Rovelli, F. Santanastasio, P. Traczyk, N. Amapane, R. Arcidiacono, S. Argiro, M. Arneodo, R. Bellan, C. Biino, N. Cartiglia, M. Costa, R. Covarelli, A. Degano, N. Demaria, L. Finco, B. Kiani, C. Mariotti, S. Maselli, E. Migliore, V. Monaco, E. Monteil, M. M. Obertino, L. Pacher, N. Pastrone, M. Pelliccioni, G. L. Pinna Angioni, F. Ravera, A. Romero, M. Ruspa, R. Sacchi, A. Solano, A. Staiano, U. Tamponi, S. Belforte, V. Candelise, M. Casarsa, F. Cossutti, G. Della Ricca, B. Gobbo, C. La Licata, M. Marone, A. Schizzi, A. Zanetti, T. A. Kropivnitskaya, S. K. Nam, D. H. Kim, G. N. Kim, M. S. Kim, D. J. Kong, S. Lee, Y. D. Oh, A. Sakharov, D. C. Son, J. A. Brochero Cifuentes, H. Kim, T. J. Kim, S. Song, S. Choi, Y. Go, D. Gyun, B. Hong, M. Jo, H. Kim, Y. Kim, B. Lee, K. Lee, K. S. Lee, S. Lee, S. K. Park, Y. Roh, H. D. Yoo, M. Choi, H. Kim, J. H. Kim, J. S. H. Lee, I. C. Park, G. Ryu, M. S. Ryu, Y. Choi, J. Goh, D. Kim, E. Kwon, J. Lee, I. Yu, V. Dudenas, A. Juodagalvis, J. Vaitkus, I. Ahmed, Z. A. Ibrahim, J. R. Komaragiri, M. A. B. Md Ali, F. Mohamad Idris, W. A. T. Wan Abdullah, M. N. Yusli, E. Casimiro Linares, H. Castilla-Valdez, E. De La Cruz-Burelo, I. Heredia-De La Cruz, A. Hernandez-Almada, R. Lopez-Fernandez, A. Sanchez-Hernandez, S. Carrillo Moreno, F. Vazquez Valencia, I. Pedraza, H. A. Salazar Ibarguen, A. Morelos Pineda, D. Krofcheck, P. H. Butler, A. Ahmad, M. Ahmad, Q. Hassan, H. R. Hoorani, W. A. Khan, T. Khurshid, M. Shoaib, H. Bialkowska, M. Bluj, B. Boimska, T. Frueboes, M. Górski, M. Kazana, K. Nawrocki, K. Romanowska-Rybinska, M. Szleper, P. Zalewski, G. Brona, K. Bunkowski, A. Byszuk, K. Doroba, A. Kalinowski, M. Konecki, J. Krolikowski, M. Misiura, M. Olszewski, M. Walczak, P. Bargassa, C. Beir ao Da Cruz E Silva, A. Di Francesco, P. Faccioli, P. G. Ferreira Parracho, M. Gallinaro, N. Leonardo, L. Lloret Iglesias, F. Nguyen, J. Rodrigues Antunes, J. Seixas, O. Toldaiev, D. Vadruccio, J. Varela, P. Vischia, S. Afanasiev, P. Bunin, M. Gavrilenko, I. Golutvin, I. Gorbunov, A. Kamenev, V. Karjavin, V. Konoplyanikov, A. Lanev, A. Malakhov, V. Matveev, P. Moisenz, V. Palichik, V. Perelygin, M. Savina, S. Shmatov, S. Shulha, N. Skatchkov, V. Smirnov, A. Zarubin, V. Golovtsov, Y. Ivanov, V. Kim, E. Kuznetsova, P. Levchenko, V. Murzin, V. Oreshkin, I. Smirnov, V. Sulimov, L. Uvarov, S. Vavilov, A. Vorobyev, Yu. Andreev, A. Dermenev, S. Gninenko, N. Golubev, A. Karneyeu, M. Kirsanov, N. Krasnikov, A. Pashenkov, D. Tlisov, A. Toropin, V. Epshteyn, V. Gavrilov, N. Lychkovskaya, V. Popov, l. Pozdnyakov, G. Safronov, A. Spiridonov, E. Vlasov, A. Zhokin, A. Bylinkin, V. Andreev, M. Azarkin, I. Dremin, M. Kirakosyan, A. Leonidov, G. Mesyats, S. V. Rusakov, A. Baskakov, A. Belyaev, E. Boos, V. Bunichev, M. Dubinin, L. Dudko, A. Ershov, A. Gribushin, V. Klyukhin, O. Kodolova, I. Lokhtin, I. Myagkov, S. Obraztsov, V. Savrin, A Snigirev, I. Azhgirey, I. Bayshev, S. Bitioukov, V. Kachanov, A. Kalinin, D. Konstantinov, V. Krychkine, V. Petrov, R. Ryutin, A. Sobol, L. Tourtchanovitch, S. Troshin, N. Tyurin, A. Uzunian, A. Volkov, P. Adzic, J. Milosevic, V. Rekovic, J. Alcaraz Maestre, E. Calvo, M. Cerrada, M. Chamizo Llatas, N. Colino, B. De La Cruz, A. Delgado Peris, D. Domínguez Vázquez, A. Escalante Del Valle, C. Fernandez Bedoya, J. P. Fernández Ramos, J. Flix, M. C. Fouz, P. Garcia-Abia, O. Gonzalez Lopez, S. Goy Lopez, J. M. Hernandez, M. I. Josa, E. Navarro De Martino, A. Pérez-Calero Yzquierdo, J. Puerta Pelayo, A. Quintario Olmeda, I. Redondo, L. Romero, J. Santaolalla, M. S. Soares, C. Albajar, J. F. de Trocóniz, M. Missiroli, D. Moran, J. Cuevas, J. Fernandez Menendez, S. Folgueras, I. Gonzalez Caballero, E. Palencia Cortezon, J. M. Vizan Garcia, I. J. Cabrillo, A. Calderon, J. R. Castiñeiras De Saa, P. De Castro Manzano, J. Duarte Campderros, M. Fernandez, J. Garcia-Ferrero, G. Gomez, A. Lopez Virto, J. Marco, R. Marco, C. Martinez Rivero, F. Matorras, F. J. Munoz Sanchez, J. Piedra Gomez, T. Rodrigo, A. Y. Rodríguez-Marrero, A. Ruiz-Jimeno, L. Scodellaro, N. Trevisani, I. Vila, R. Vilar Cortabitarte, D. Abbaneo, E. Auffray, G. Auzinger, M. Bachtis, P. Baillon, A. H. Ball, D. Barney, A. Benaglia, J. Bendavid, L. Benhabib, J. F. Benitez, G. M. Berruti, P. Bloch, A. Bocci, A. Bonato, C. Botta, H. Breuker, T. Camporesi, R. Castello, G. Cerminara, M. D’Alfonso, D. d’Enterria, A. Dabrowski, V. Daponte, A. David, M. De Gruttola, F. De Guio, A. De Roeck, S. De Visscher, E. Di Marco, M. Dobson, M. Dordevic, B. Dorney, T. du Pree, M. Dünser, N. Dupont, A. Elliott-Peisert, G. Franzoni, W. Funk, D. Gigi, K. Gill, D. Giordano, M. Girone, F. Glege, R. Guida, S. Gundacker, M. Guthoff, J. Hammer, P. Harris, J. Hegeman, V. Innocente, P. Janot, H. Kirschenmann, M. J. Kortelainen, K. Kousouris, K. Krajczar, P. Lecoq, C. Lourenço, M. T. Lucchini, N. Magini, L. Malgeri, M. Mannelli, A. Martelli, L. Masetti, F. Meijers, S. Mersi, E. Meschi, F. Moortgat, S. Morovic, M. Mulders, M. V. Nemallapudi, H. Neugebauer, S. Orfanelli, L. Orsini, L. Pape, E. Perez, M. Peruzzi, A. Petrilli, G. Petrucciani, A. Pfeiffer, D. Piparo, A. Racz, G. Rolandi, M. Rovere, M. Ruan, H. Sakulin, C. Schäfer, C. Schwick, M Seidel, A. Sharma, P. Silva, M. Simon, P. Sphicas, J. Steggemann, B. Stieger, M. Stoye, Y. Takahashi, D. Treille, A. Triossi, A. Tsirou, G. I. Veres, N. Wardle, H. K. Wöhri, A. Zagozdzinska, W. D. Zeuner, W. Bertl, K. Deiters, W. Erdmann, R. Horisberger, Q. Ingram, H. C. Kaestli, D. Kotlinski, U. Langenegger, D. Renker, T. Rohe, F. Bachmair, L. Bäni, L. Bianchini, B. Casal, G. Dissertori, M. Dittmar, M. Donegà, P. Eller, C. Grab, C. Heidegger, D. Hits, J. Hoss, G. Kasieczka, W. Lustermann, B. Mangano, M. Marionneau, P. Martinez Ruiz del Arbol, M. Masciovecchio, D. Meister, F. Micheli, P. Musella, F. Nessi-Tedaldi, F. Pandolfi, J. Pata, F. Pauss, L. Perrozzi, M. Quittnat, M. Rossini, A. Starodumov, M. Takahashi, V. R. Tavolaro, K. Theofilatos, R. Wallny, T. K. Aarrestad, C. Amsler, L. Caminada, M. F. Canelli, V. Chiochia, A. De Cosa, C. Galloni, A. Hinzmann, T. Hreus, B. Kilminster, C. Lange, J. Ngadiuba, D. Pinna, P. Robmann, F. J. Ronga, D. Salerno, Y. Yang, M. Cardaci, K. H. Chen, T. H. Doan, Sh. Jain, R. Khurana, M. Konyushikhin, C. M. Kuo, W. Lin, Y. J. Lu, S. S. Yu, Arun Kumar, R. Bartek, P. Chang, Y. H. Chang, Y. W. Chang, Y. Chao, K. F. Chen, P. H. Chen, C. Dietz, F. Fiori, U. Grundler, W.-S. Hou, Y. Hsiung, Y. F. Liu, R.-S. Lu, M. Miñano Moya, E. Petrakou, J. f. Tsai, Y. M. Tzeng, B. Asavapibhop, K. Kovitanggoon, G. Singh, N. Srimanobhas, N. Suwonjandee, A. Adiguzel, M. N. Bakirci, S. Cerci, Z. S. Demiroglu, C. Dozen, I. Dumanoglu, E. Eskut, S. Girgis, G. Gokbulut, Y. Guler, E. Gurpinar, I. Hos, E. E. Kangal, A. Kayis Topaksu, G. Onengut, K. Ozdemir, A. Polatoz, M. Vergili, C. Zorbilmez, I. V. Akin, B. Bilin, S. Bilmis, B. Isildak, G. Karapinar, M. Yalvac, M. Zeyrek, E. Gülmez, M. Kaya, O. Kaya, E. A. Yetkin, T. Yetkin, A. Cakir, K. Cankocak, S. Sen, F. I. Vardarlı, B. Grynyov, L. Levchuk, P. Sorokin, R. Aggleton, F. Ball, L. Beck, J. J. Brooke, E. Clement, D. Cussans, H. Flacher, J. Goldstein, M. Grimes, G. P. Heath, H. F. Heath, J. Jacob, L. Kreczko, C. Lucas, Z. Meng, D. M. Newbold, S. Paramesvaran, A. Poll, T. Sakuma, S. Seif El Nasr-storey, S. Senkin, D. Smith, V. J. Smith, K. W. Bell, A. Belyaev, C. Brew, R. M. Brown, L. Calligaris, D. Cieri, D. J. A. Cockerill, J. A. Coughlan, K. Harder, S. Harper, E. Olaiya, D. Petyt, C. H. Shepherd-Themistocleous, A. Thea, I. R. Tomalin, T. Williams, W. J. Womersley, S. D. Worm, M. Baber, R. Bainbridge, O. Buchmuller, A. Bundock, D. Burton, S. Casasso, M. Citron, D. Colling, L. Corpe, N. Cripps, P. Dauncey, G. Davies, A. De Wit, M. Della Negra, P. Dunne, A. Elwood, W. Ferguson, J. Fulcher, D. Futyan, G. Hall, G. Iles, M. Kenzie, R. Lane, R. Lucas, L. Lyons, A.-M. Magnan, S. Malik, J. Nash, A. Nikitenko, J. Pela, M. Pesaresi, K. Petridis, D. M. Raymond, A. Richards, A. Rose, C. Seez, A. Tapper, K. Uchida, M. Vazquez Acosta, T. Virdee, S. C. Zenz, J. E. Cole, P. R. Hobson, A. Khan, P. Kyberd, D. Leggat, D. Leslie, I. D. Reid, P. Symonds, L. Teodorescu, M. Turner, A. Borzou, K. Call, J. Dittmann, K. Hatakeyama, H. Liu, N. Pastika, O. Charaf, S. I. Cooper, C. Henderson, P. Rumerio, D. Arcaro, A. Avetisyan, T. Bose, C. Fantasia, D. Gastler, P. Lawson, D. Rankin, C. Richardson, J. Rohlf, J. St. John, L. Sulak, D. Zou, J. Alimena, E. Berry, S. Bhattacharya, D. Cutts, N. Dhingra, A. Ferapontov, A. Garabedian, J. Hakala, U. Heintz, E. Laird, G. Landsberg, Z. Mao, M. Narain, S. Piperov, S. Sagir, R. Syarif, R. Breedon, G. Breto, M. Calderon De La Barca Sanchez, S. Chauhan, M. Chertok, J. Conway, R. Conway, P. T. Cox, R. Erbacher, M. Gardner, W. Ko, R. Lander, M. Mulhearn, D. Pellett, J. Pilot, F. Ricci-Tam, S. Shalhout, J. Smith, M. Squires, D. Stolp, M. Tripathi, S. Wilbur, R. Yohay, R. Cousins, P. Everaerts, C. Farrell, J. Hauser, M. Ignatenko, D. Saltzberg, E. Takasugi, V. Valuev, M. Weber, K. Burt, R. Clare, J. Ellison, J. W. Gary, G. Hanson, J. Heilman, M. Ivova PANEVA, P. Jandir, E. Kennedy, F. Lacroix, O. R. Long, A. Luthra, M. Malberti, M. Olmedo Negrete, A. Shrinivas, H. Wei, S. Wimpenny, B. R. Yates, J. G. Branson, G. B. Cerati, S. Cittolin, R. T. D’Agnolo, M Derdzinski, A. Holzner, R. Kelley, D. Klein, J. Letts, I. Macneill, D. Olivito, S. Padhi, M. Pieri, M. Sani, V. Sharma, S. Simon, M. Tadel, A. Vartak, S. Wasserbaech, C. Welke, F. Würthwein, A. Yagil, G. Zevi Della Porta, J. Bradmiller-Feld, C. Campagnari, A. Dishaw, V. Dutta, K. Flowers, M. Franco Sevilla, P. Geffert, C. George, F. Golf, L. Gouskos, J. Gran, J. Incandela, N. Mccoll, S. D. Mullin, J. Richman, D. Stuart, I. Suarez, C. West, J. Yoo, D. Anderson, A. Apresyan, A. Bornheim, J. Bunn, Y. Chen, J. Duarte, A. Mott, H. B. Newman, C. Pena, M. Pierini, M. Spiropulu, J. R. Vlimant, S. Xie, R. Y. Zhu, M. B. Andrews, V. Azzolini, A. Calamba, B. Carlson, T. Ferguson, M. Paulini, J. Russ, M. Sun, H. Vogel, I. Vorobiev, J. P. Cumalat, W. T. Ford, A. Gaz, F. Jensen, A. Johnson, M. Krohn, T. Mulholland, U. Nauenberg, K. Stenson, S. R. Wagner, J. Alexander, A. Chatterjee, J. Chaves, J. Chu, S. Dittmer, N. Eggert, N. Mirman, G. Nicolas Kaufman, J. R. Patterson, A. Rinkevicius, A. Ryd, L. Skinnari, L. Soffi, W. Sun, S. M. Tan, W. D. Teo, J. Thom, J. Thompson, J. Tucker, Y. Weng, P. Wittich, S. Abdullin, M. Albrow, J. Anderson, G. Apollinari, S. Banerjee, L. A. T. Bauerdick, A. Beretvas, J. Berryhill, P. C. Bhat, G. Bolla, K. Burkett, J. N. Butler, H. W. K. Cheung, F. Chlebana, S. Cihangir, V. D. Elvira, I. Fisk, J. Freeman, E. Gottschalk, L. Gray, D. Green, S. Grünendahl, O. Gutsche, J. Hanlon, D. Hare, R. M. Harris, S. Hasegawa, J. Hirschauer, Z. Hu, B. Jayatilaka, S. Jindariani, M. Johnson, U. Joshi, A. W. Jung, B. Klima, B. Kreis, S. Kwan, S. Lammel, J. Linacre, D. Lincoln, R. Lipton, T. Liu, R. Lopes De Sá, J. Lykken, K. Maeshima, J. M. Marraffino, V. I. Martinez Outschoorn, S. Maruyama, D. Mason, P. McBride, P. Merkel, K. Mishra, S. Mrenna, S. Nahn, C. Newman-Holmes, V. O’Dell, K. Pedro, O. Prokofyev, G. Rakness, E. Sexton-Kennedy, A. Soha, W. J. Spalding, L. Spiegel, N. Strobbe, L. Taylor, S. Tkaczyk, N. V. Tran, L. Uplegger, E. W. Vaandering, C. Vernieri, M. Verzocchi, R. Vidal, H. A. Weber, A. Whitbeck, F. Yang, D. Acosta, P. Avery, P. Bortignon, D. Bourilkov, A. Carnes, M. Carver, D. Curry, S. Das, R. D. Field, I. K. Furic, S. V. Gleyzer, J. Hugon, J. Konigsberg, A. Korytov, J. F. Low, P. Ma, K. Matchev, H. Mei, P. Milenovic, G. Mitselmakher, D. Rank, R. Rossin, L. Shchutska, M. Snowball, D. Sperka, N. Terentyev, L. Thomas, J. Wang, S. Wang, J. Yelton, S. Hewamanage, S. Linn, P. Markowitz, G. Martinez, J. L. Rodriguez, A. Ackert, J. R. Adams, T. Adams, A. Askew, J. Bochenek, B. Diamond, J. Haas, S. Hagopian, V. Hagopian, K. F. Johnson, A. Khatiwada, H. Prosper, M. Weinberg, M. M. Baarmand, V. Bhopatkar, S. Colafranceschi, M. Hohlmann, H. Kalakhety, D. Noonan, T. Roy, F. Yumiceva, M. R. Adams, L. Apanasevich, D. Berry, R. R. Betts, I. Bucinskaite, R. Cavanaugh, O. Evdokimov, L. Gauthier, C. E. Gerber, D. J. Hofman, P. Kurt, C. O’Brien, l. D. Sandoval Gonzalez, C. Silkworth, P. Turner, N. Varelas, Z. Wu, M. Zakaria, B. Bilki, W. Clarida, K. Dilsiz, S. Durgut, R. P. Gandrajula, M. Haytmyradov, V. Khristenko, J.-P. Merlo, H. Mermerkaya, A. Mestvirishvili, A. Moeller, J. Nachtman, H. Ogul, Y. Onel, F. Ozok, A. Penzo, C. Snyder, E. Tiras, J. Wetzel, K. Yi, I. Anderson, B. A. Barnett, B. Blumenfeld, N. Eminizer, D. Fehling, L. Feng, A. V. Gritsan, P. Maksimovic, C. Martin, M. Osherson, J. Roskes, A. Sady, U. Sarica, M. Swartz, M. Xiao, Y. Xin, C. You, P. Baringer, A. Bean, G. Benelli, C. Bruner, R. P. Kenny, D. Majumder, M. Malek, M. Murray, S. Sanders, R. Stringer, Q. Wang, A. Ivanov, K. Kaadze, S. Khalil, M. Makouski, Y. Maravin, A. Mohammadi, L. K. Saini, N. Skhirtladze, S. Toda, D. Lange, F. Rebassoo, D. Wright, C. Anelli, A. Baden, O. Baron, A. Belloni, B. Calvert, S. C. Eno, C. Ferraioli, J. A. Gomez, N. J. Hadley, S. Jabeen, R. G. Kellogg, T. Kolberg, J. Kunkle, Y. Lu, A. C. Mignerey, Y. H. Shin, A. Skuja, M. B. Tonjes, S. C. Tonwar, A. Apyan, R. Barbieri, A. Baty, K. Bierwagen, S. Brandt, W. Busza, I. A. Cali, Z. Demiragli, L. Di Matteo, G. Gomez Ceballos, M. Goncharov, D. Gulhan, Y. Iiyama, G. M. Innocenti, M. Klute, D. Kovalskyi, Y. S. Lai, Y.-J. Lee, A. Levin, P. D. Luckey, A. C. Marini, C. Mcginn, C. Mironov, S. Narayanan, X. Niu, C. Paus, D. Ralph, C. Roland, G. Roland, J. Salfeld-Nebgen, G. S. F. Stephans, K. Sumorok, M. Varma, D. Velicanu, J. Veverka, J. Wang, T. W. Wang, B. Wyslouch, M. Yang, V. Zhukova, B. Dahmes, A. Evans, A. Finkel, A. Gude, P. Hansen, S. Kalafut, S. C. Kao, K. Klapoetke, Y. Kubota, Z. Lesko, J. Mans, S. Nourbakhsh, N. Ruckstuhl, R. Rusack, N. Tambe, J. Turkewitz, J. G. Acosta, S. Oliveros, E. Avdeeva, K. Bloom, S. Bose, D. R. Claes, A. Dominguez, C. Fangmeier, R. Gonzalez Suarez, R. Kamalieddin, J. Keller, D. Knowlton, I. Kravchenko, F. Meier, J. Monroy, F. Ratnikov, J. E. Siado, G. R. Snow, M. Alyari, J. Dolen, J. George, A. Godshalk, C. Harrington, I. Iashvili, J. Kaisen, A. Kharchilava, A. Kumar, S. Rappoccio, B. Roozbahani, G. Alverson, E. Barberis, D. Baumgartel, M. Chasco, A. Hortiangtham, A. Massironi, D. M. Morse, D. Nash, T. Orimoto, R. Teixeira De Lima, D. Trocino, R.-J. Wang, D. Wood, J. Zhang, K. A. Hahn, A. Kubik, N. Mucia, N. Odell, B. Pollack, A. Pozdnyakov, M. Schmitt, S. Stoynev, K. Sung, M. Trovato, M. Velasco, A. Brinkerhoff, N. Dev, M. Hildreth, C. Jessop, D. J. Karmgard, N. Kellams, K. Lannon, S. Lynch, N. Marinelli, F. Meng, C. Mueller, Y. Musienko, T. Pearson, M. Planer, A. Reinsvold, R. Ruchti, G. Smith, S. Taroni, N. Valls, M. Wayne, M. Wolf, A. Woodard, L. Antonelli, J. Brinson, B. Bylsma, L. S. Durkin, S. Flowers, A. Hart, C. Hill, R. Hughes, W. Ji, K. Kotov, T. Y. Ling, B. Liu, W. Luo, D. Puigh, M. Rodenburg, B. L. Winer, H. W. Wulsin, O. Driga, P. Elmer, J. Hardenbrook, P. Hebda, S. A. Koay, P. Lujan, D. Marlow, T. Medvedeva, M. Mooney, J. Olsen, C. Palmer, P. Piroué, H. Saka, D. Stickland, C. Tully, A. Zuranski, S. Malik, V. E. Barnes, D. Benedetti, D. Bortoletto, L. Gutay, M. K. Jha, M. Jones, K. Jung, D. H. Miller, N. Neumeister, B. C. Radburn-Smith, X. Shi, I. Shipsey, D. Silvers, J. Sun, A. Svyatkovskiy, F. Wang, W. Xie, L. Xu, N. Parashar, J. Stupak, A. Adair, B. Akgun, Z. Chen, K. M. Ecklund, F. J. M. Geurts, M. Guilbaud, W. Li, B. Michlin, M. Northup, B. P. Padley, R. Redjimi, J. Roberts, J. Rorie, Z. Tu, J. Zabel, B. Betchart, A. Bodek, P. de Barbaro, R. Demina, Y. Eshaq, T. Ferbel, M. Galanti, A. Garcia-Bellido, J. Han, A. Harel, O. Hindrichs, A. Khukhunaishvili, G. Petrillo, P. Tan, M. Verzetti, S. Arora, A. Barker, J. P. Chou, C. Contreras-Campana, E. Contreras-Campana, D. Duggan, D. Ferencek, Y. Gershtein, R. Gray, E. Halkiadakis, D. Hidas, E. Hughes, S. Kaplan, R. Kunnawalkam Elayavalli, A. Lath, K. Nash, S. Panwalkar, M. Park, S. Salur, S. Schnetzer, D. Sheffield, S. Somalwar, R. Stone, S. Thomas, P. Thomassen, M. Walker, M. Foerster, G. Riley, K. Rose, S. Spanier, A. York, O. Bouhali, A. Castaneda Hernandez, M. Dalchenko, M. De Mattia, A. Delgado, S. Dildick, R. Eusebi, J. Gilmore, T. Kamon, V. Krutelyov, R. Mueller, I. Osipenkov, Y. Pakhotin, R. Patel, A. Perloff, A. Rose, A. Safonov, A. Tatarinov, K. A. Ulmer, N. Akchurin, C. Cowden, J. Damgov, C. Dragoiu, P. R. Dudero, J. Faulkner, S. Kunori, K. Lamichhane, S. W. Lee, T. Libeiro, S. Undleeb, I. Volobouev, E. Appelt, A. G. Delannoy, S. Greene, A. Gurrola, R. Janjam, W. Johns, C. Maguire, Y. Mao, A. Melo, H. Ni, P. Sheldon, B. Snook, S. Tuo, J. Velkovska, Q. Xu, M. W. Arenton, B. Cox, B. Francis, J. Goodell, R. Hirosky, A. Ledovskoy, H. Li, C. Lin, C. Neu, T Sinthuprasith, X. Sun, Y. Wang, E. Wolfe, J. Wood, F. Xia, C. Clarke, R. Harr, P. E. Karchin, C. Kottachchi Kankanamge Don, P. Lamichhane, J. Sturdy, D. A. Belknap, D. Carlsmith, M. Cepeda, S. Dasu, L. Dodd, S. Duric, B. Gomber, M. Grothe, R. Hall-Wilton, M. Herndon, A. Hervé, P. Klabbers, A. Lanaro, A. Levine, K. Long, R. Loveless, A. Mohapatra, I. Ojalvo, T. Perry, G. A. Pierro, G. Polese, T. Ruggles, T. Sarangi, A. Savin, A. Sharma, N. Smith, W. H. Smith, D. Taylor, N. Woods, The CMS Collaboration

**Affiliations:** 1 Yerevan Physics Institute, Yerevan, Armenia; 2 Institut für Hochenergiephysik der OeAW, Vienna, Austria; 3 National Centre for Particle and High Energy Physics, Minsk, Belarus; 4 Universiteit Antwerpen, Antwerp, Belgium; 5 Vrije Universiteit Brussel, Brussels, Belgium; 6 Université Libre de Bruxelles, Brussels, Belgium; 7 Ghent University, Ghent, Belgium; 8 Université Catholique de Louvain, Louvain-la-Neuve, Belgium; 9 Université de Mons, Mons, Belgium; 10 Centro Brasileiro de Pesquisas Fisicas, Rio de Janeiro, Brazil; 11 Universidade do Estado do Rio de Janeiro, Rio de Janeiro, Brazil; 12 Universidade Estadual Paulista, Universidade Federal do ABC, São Paulo, Brazil; 13 Institute for Nuclear Research and Nuclear Energy, Sofia, Bulgaria; 14 University of Sofia, Sofia, Bulgaria; 15 Institute of High Energy Physics, Beijing, China; 16 State Key Laboratory of Nuclear Physics and Technology, Peking University, Beijing, China; 17 Universidad de Los Andes, Bogotá, Colombia; 18 Faculty of Electrical Engineering, Mechanical Engineering and Naval Architecture, University of Split, Split, Croatia; 19 Faculty of Science, University of Split, Split, Croatia; 20 Institute Rudjer Boskovic, Zagreb, Croatia; 21 University of Cyprus, Nicosia, Cyprus; 22 Charles University, Prague, Czech Republic; 23 Academy of Scientific Research and Technology of the Arab Republic of Egypt, Egyptian Network of High Energy Physics, Cairo, Egypt; 24 National Institute of Chemical Physics and Biophysics, Tallinn, Estonia; 25 Department of Physics, University of Helsinki, Helsinki, Finland; 26 Helsinki Institute of Physics, Helsinki, Finland; 27 Lappeenranta University of Technology, Lappeenranta, Finland; 28 DSM/IRFU, CEA/Saclay, Gif-sur-Yvette, France; 29 Laboratoire Leprince-Ringuet, Ecole Polytechnique, IN2P3-CNRS, Palaiseau, France; 30 Institut Pluridisciplinaire Hubert Curien, Université de Strasbourg, Université de Haute Alsace Mulhouse, CNRS/IN2P3, Strasbourg, France; 31 Centre de Calcul de l’Institut National de Physique Nucleaire et de Physique des Particules, CNRS/IN2P3, Villeurbanne, France; 32 Institut de Physique Nucléaire de Lyon, Université de Lyon, Université Claude Bernard Lyon 1, CNRS-IN2P3, Villeurbanne, France; 33 Georgian Technical University, Tbilisi, Georgia; 34 Tbilisi State University, Tbilisi, Georgia; 35 I. Physikalisches Institut, RWTH Aachen University, Aachen, Germany; 36 III. Physikalisches Institut A, RWTH Aachen University, Aachen, Germany; 37 III. Physikalisches Institut B, RWTH Aachen University, Aachen, Germany; 38 Deutsches Elektronen-Synchrotron, Hamburg, Germany; 39 University of Hamburg, Hamburg, Germany; 40 Institut für Experimentelle Kernphysik, Karlsruhe, Germany; 41 Institute of Nuclear and Particle Physics (INPP), NCSR Demokritos, Aghia Paraskevi, Greece; 42 National and Kapodistrian, University of Athens, Athens, Greece; 43 University of Ioánnina, Ioannina, Greece; 44 Wigner Research Centre for Physics, Budapest, Hungary; 45 Institute of Nuclear Research ATOMKI, Debrecen, Hungary; 46 University of Debrecen, Debrecen, Hungary; 47 National Institute of Science Education and Research, Bhubaneswar, India; 48 Panjab University, Chandigarh, India; 49 University of Delhi, Delhi, India; 50 Saha Institute of Nuclear Physics, Kolkata, India; 51 Bhabha Atomic Research Centre, Mumbai, India; 52 Tata Institute of Fundamental Research, Mumbai, India; 53 Indian Institute of Science Education and Research (IISER), Pune, India; 54 Institute for Research in Fundamental Sciences (IPM), Tehran, Iran; 55 University College Dublin, Dublin, Ireland; 56 INFN Sezione di Bari, Università di Bari, Politecnico di Bari, Bari, Italy; 57 INFN Sezione di Bologna, Università di Bologna, Bologna, Italy; 58 INFN Sezione di Catania, Università di Catania, Catania, Italy; 59 INFN Sezione di Firenze, Università di Firenze, Florence, Italy; 60 INFN Laboratori Nazionali di Frascati, Frascati, Italy; 61 INFN Sezione di Genova, Università di Genova, Genoa, Italy; 62 INFN Sezione di Milano-Bicocca, Università di Milano-Bicocca, Milan, Italy; 63 INFN Sezione di Napoli, Università di Napoli ‘Federico II’, Napoli, Italy, Università della Basilicata, Potenza, Italy, Università G. Marconi, Rome, Italy; 64 INFN Sezione di Padova, Università di Padova, Padova, Italy, Università di Trento, Trento, Italy; 65 INFN Sezione di Pavia, Università di Pavia, Pavia, Italy; 66 INFN Sezione di Perugia, Università di Perugia, Perugia, Italy; 67 INFN Sezione di Pisa, Università di Pisa, Scuola Normale Superiore di Pisa, Pisa, Italy; 68 INFN Sezione di Roma, Università di Roma, Rome, Italy; 69 INFN Sezione di Torino, Università di Torino, Turin, Italy, Università del Piemonte Orientale, Novara, Italy; 70 INFN Sezione di Trieste, Università di Trieste, Trieste, Italy; 71 Kangwon National University, Chunchon, Korea; 72 Kyungpook National University, Daegu, Korea; 73 Chonbuk National University, Jeonju, Korea; 74 Institute for Universe and Elementary Particles, Chonnam National University, Kwangju, Korea; 75 Korea University, Seoul, Korea; 76 Seoul National University, Seoul, Korea; 77 University of Seoul, Seoul, Korea; 78 Sungkyunkwan University, Suwon, Korea; 79 Vilnius University, Vilnius, Lithuania; 80 National Centre for Particle Physics, Universiti Malaya, Kuala Lumpur, Malaysia; 81 Centro de Investigacion y de Estudios Avanzados del IPN, Mexico City, Mexico; 82 Universidad Iberoamericana, Mexico City, Mexico; 83 Benemerita Universidad Autonoma de Puebla, Puebla, Mexico; 84 Universidad Autónoma de San Luis Potosí, San Luis Potosí, Mexico; 85 University of Auckland, Auckland, New Zealand; 86 University of Canterbury, Christchurch, New Zealand; 87 National Centre for Physics, Quaid-I-Azam University, Islamabad, Pakistan; 88 National Centre for Nuclear Research, Swierk, Poland; 89 Institute of Experimental Physics, Faculty of Physics, University of Warsaw, Warsaw, Poland; 90 Laboratório de Instrumentação e Física Experimental de Partículas, Lisbon, Portugal; 91 Joint Institute for Nuclear Research, Dubna, Russia; 92 Petersburg Nuclear Physics Institute, Gatchina, St. Petersburg, Russia; 93 Institute for Nuclear Research, Moscow, Russia; 94 Institute for Theoretical and Experimental Physics, Moscow, Russia; 95 National Research Nuclear University ‘Moscow Engineering Physics Institute’ (MEPhI), Moscow, Russia; 96 P. N. Lebedev Physical Institute, Moscow, Russia; 97 Skobeltsyn Institute of Nuclear Physics, Lomonosov Moscow State University, Moscow, Russia; 98 State Research Center of Russian Federation, Institute for High Energy Physics, Protvino, Russia; 99 Faculty of Physics and Vinca Institute of Nuclear Sciences, University of Belgrade, Belgrade, Serbia; 100 Centro de Investigaciones Energéticas Medioambientales y Tecnológicas (CIEMAT), Madrid, Spain; 101 Universidad Autónoma de Madrid, Madrid, Spain; 102 Universidad de Oviedo, Oviedo, Spain; 103 Instituto de Física de Cantabria (IFCA), CSIC-Universidad de Cantabria, Santander, Spain; 104 CERN, European Organization for Nuclear Research, Geneva, Switzerland; 105 Paul Scherrer Institut, Villigen, Switzerland; 106 Institute for Particle Physics, ETH Zurich, Zurich, Switzerland; 107 Universität Zürich, Zurich, Switzerland; 108 National Central University, Chung-Li, Taiwan; 109 National Taiwan University (NTU), Taipei, Taiwan; 110 Department of Physics, Faculty of Science, Chulalongkorn University, Bangkok, Thailand; 111 Cukurova University, Adana, Turkey; 112 Physics Department, Middle East Technical University, Ankara, Turkey; 113 Bogazici University, Istanbul, Turkey; 114 Istanbul Technical University, Istanbul, Turkey; 115 Institute for Scintillation Materials of National Academy of Science of Ukraine, Kharkov, Ukraine; 116 National Scientific Center, Kharkov Institute of Physics and Technology, Kharkov, Ukraine; 117 University of Bristol, Bristol, UK; 118 Rutherford Appleton Laboratory, Didcot, UK; 119 Imperial College, London, UK; 120 Brunel University, Uxbridge, UK; 121 Baylor University, Waco, USA; 122 The University of Alabama, Tuscaloosa, USA; 123 Boston University, Boston, USA; 124 Brown University, Providence, USA; 125 University of California, Davis, Davis, USA; 126 University of California, Los Angeles, USA; 127 University of California, Riverside, Riverside, USA; 128 University of California, San Diego, La Jolla, USA; 129 University of California, Santa Barbara, Santa Barbara, USA; 130 California Institute of Technology, Pasadena, USA; 131 Carnegie Mellon University, Pittsburgh, USA; 132 University of Colorado Boulder, Boulder, USA; 133 Cornell University, Ithaca, USA; 134 Fermi National Accelerator Laboratory, Batavia, USA; 135 University of Florida, Gainesville, USA; 136 Florida International University, Miami, USA; 137 Florida State University, Tallahassee, USA; 138 Florida Institute of Technology, Melbourne, USA; 139 University of Illinois at Chicago (UIC), Chicago, USA; 140 The University of Iowa, Iowa City, USA; 141 Johns Hopkins University, Baltimore, USA; 142 The University of Kansas, Lawrence, USA; 143 Kansas State University, Manhattan, USA; 144 Lawrence Livermore National Laboratory, Livermore, USA; 145 University of Maryland, College Park, USA; 146 Massachusetts Institute of Technology, Cambridge, USA; 147 University of Minnesota, Minneapolis, USA; 148 University of Mississippi, Oxford, USA; 149 University of Nebraska-Lincoln, Lincoln, USA; 150 State University of New York at Buffalo, Buffalo, USA; 151 Northeastern University, Boston, USA; 152 Northwestern University, Evanston, USA; 153 University of Notre Dame, Notre Dame, USA; 154 The Ohio State University, Columbus, USA; 155 Princeton University, Princeton, USA; 156 University of Puerto Rico, Mayaguez, USA; 157 Purdue University, West Lafayette, USA; 158 Purdue University Calumet, Hammond, USA; 159 Rice University, Houston, USA; 160 University of Rochester, Rochester, USA; 161 Rutgers, The State University of New Jersey, Piscataway, USA; 162 University of Tennessee, Knoxville, USA; 163 Texas A&M University, College Station, USA; 164 Texas Tech University, Lubbock, USA; 165 Vanderbilt University, Nashville, USA; 166 University of Virginia, Charlottesville, USA; 167 Wayne State University, Detroit, USA; 168 University of Wisconsin-Madison, Madison, WI USA; 169 CERN, Geneva, Switzerland

## Abstract

A search for a massive resonance ${\mathrm{W}^{\prime }}$decaying into a W and a Higgs boson in the $\ell \nu \mathrm{b} \overline{\mathrm{b}}$ ($\ell = \mathrm {e}$, $\mu$) final state is presented. Results are based on data corresponding to an integrated luminosity of 19.7$\,\text {fb}^{{-1}}$ of proton–proton collisions at $\sqrt{s}=8$ $~\text {TeV}$, collected using the CMS detector at the LHC. For a high-mass ($\gtrsim$1$~\text {TeV}$) resonance, the two bottom quarks coming from the Higgs boson decay are reconstructed as a single jet, which can be tagged by placing requirements on its substructure and flavour. Exclusion limits at 95 % confidence level are set on the production cross section of a narrow resonance decaying into WH, as a function of its mass. In the context of a little Higgs model, a lower limit on the ${\mathrm{W}^{\prime }}$ mass of 1.4$~\text {TeV}$ is set. In a heavy vector triplet model that mimics the properties of composite Higgs models, a lower limit on the ${\mathrm{W}^{\prime }}$ mass of 1.5$~\text {TeV}$ is set. In the context of this model, the results are combined with related searches to obtain a lower limit on the ${\mathrm{W}^{\prime }}$ mass of 1.8$~\text {TeV}$, the most restrictive to date for decays to a pair of standard model bosons.

## Introduction

This paper presents a search for massive resonances decaying into a W and a standard model (SM) Higgs boson (H) [1–4] in the $\ell \nu \mathrm{b} {\bar{\mathrm{b}}}$ ($\ell = \mathrm {e}$, $\mu$) final state. Such processes are distinctive features of several extensions of the SM such as composite Higgs [5–7], SU(5)/SO(5) Littlest Higgs (LH) [8–11], technicolor [12, 13], and left-right symmetric models [14]. These models provide solutions to the hierarchy problem and predict new particles including additional gauge bosons such as a heavy ${\mathrm{W}^{\prime }}$. The ${\mathrm{W}^{\prime }}$ in these models can have large branching fractions to WH and WZ, while the decays to fermions can be suppressed. The recently proposed heavy vector triplet (HVT) model [15] generalizes a large class of specific models that predict new heavy spin-1 vector bosons. In this model, the resonance is described by a simplified Lagrangian in terms of a small number of parameters representing its mass and couplings to SM bosons and fermions.

For a ${\mathrm{W}^{\prime }}$ with SM couplings to fermions and thus reduced decay branching ratio to SM bosons, the most stringent limits on production cross sections are reported in searches with leptonic final states [16, 17]. The current lower limit on the ${\mathrm{W}^{\prime }}$ mass is 3.3$~\text {TeV}$. In the same context, searches for a ${\mathrm{W}^{\prime }}$ decaying into a pair of SM vector bosons (WZ) [18–21] provide a lower mass limit of 1.7$~\text {TeV}$. In the context of a HVT model with reduced couplings to fermions (HVT model B), the most stringent limit of 1.7$~\text {TeV}$ on the ${\mathrm{W}^{\prime }}$/$\mathrm{Z}^{\prime }$ mass is set by a search for ${\mathrm{W}}^\prime /{\mathrm{Z}}^\prime \rightarrow \mathrm{WH}/\mathrm{ZH} \rightarrow \mathrm{q} {\bar{\mathrm{q}}} \mathrm{b} {\bar{\mathrm{b}}}$ [22]. The same model is used to interpret the results of a search for $\mathrm{W}^\prime /\mathrm{Z}^\prime \rightarrow \mathrm{WH}/\mathrm{ZH} \rightarrow \ell \nu /\ell \ell /\nu \nu +\mathrm{b} {\bar{\mathrm{b}}}$ [23]. A lower limit on the ${\mathrm{W}^{\prime }}$ mass of 1.5$~\text {TeV}$ is set in the same final state reported in Ref. [23]. Finally, a specific search for $\mathrm{Z}^\prime \rightarrow \mathrm{ZH} \rightarrow \mathrm{q}{\bar{\mathrm{q}}} \tau ^{+}\tau ^{-}$ was reported in Ref. [24] and interpreted in the context of the same HVT model B.

**Fig. 1:**
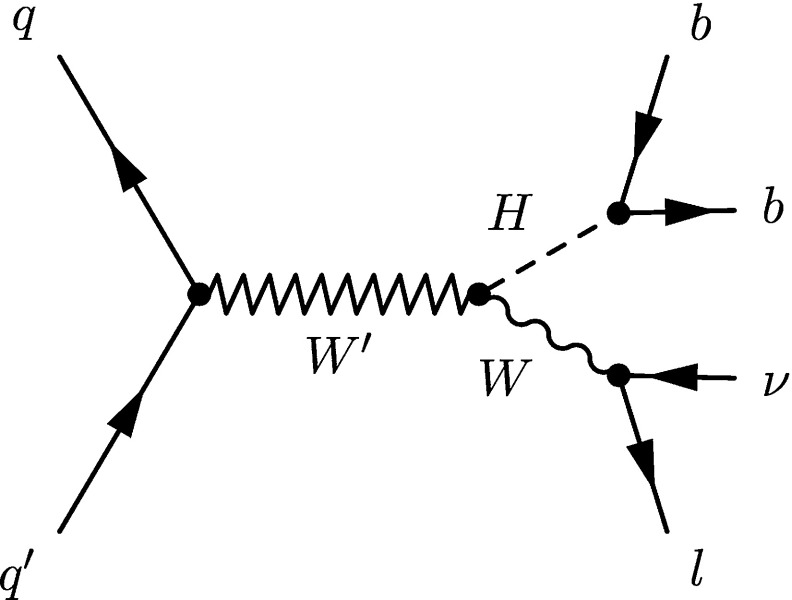
Production of a resonance decaying into WH

This analysis is based on proton–proton collision data at $\sqrt{s}=8$ TeV collected by the CMS experiment at the CERN LHC during 2012, corresponding to an integrated luminosity of 19.7$\,\text {fb}^{{-1}}$. The signal considered is the production of a resonance with mass above 0.8$~\text {TeV}$ decaying into WH, where the Higgs boson decays into a bottom quark–antiquark pair and the W boson decays into a charged lepton and a neutrino (Fig. 1). It is assumed that the resonance is narrow, i.e. that its intrinsic width is much smaller than the experimental resolution.

The search strategy is closely related to the search for high mass WW resonances in the $\ell \nu \mathrm{q} {\bar{\mathrm{q}}}$ final state, described in Ref. [25], with the addition of b tagging techniques. We search for resonances in the invariant mass of the WH system on top of a smoothly falling background distribution, where the background mainly comprises events involving pair produced top quarks ($\mathrm{t}\overline{\mathrm{t}}$) or a W boson produced in association with jets (W+jets). For the resonance mass range considered, the two quarks from the Higgs boson decay would be separated by a small angle, resulting in the detection of a single jet after hadronization. This jet is tagged as coming from a Higgs boson through the estimation of its invariant mass, application of jet substructure techniques [26], and use of specialized b tagging techniques for high transverse momentum ($p_{\mathrm {T}}$) Higgs bosons [27].

The results of this analysis are also combined with two previous results [22, 24] to obtain a further improvement in sensitivity.

## CMS detector

The central feature of the CMS apparatus is a superconducting solenoid of 6$\text {\,m}$ internal diameter, providing a field of 3.8$\text {\,T}$. Within the field volume are a silicon pixel and strip tracker, a crystal electromagnetic calorimeter (ECAL), and a brass and scintillator hadronic calorimeter (HCAL). The CMS tracker consists of 1440 silicon pixel and 15 148 silicon strip detector modules covering a pseudorapidity range of $|\eta |< 2.5$. The ECAL consists of nearly 76 000 lead tungstate crystals, which provide coverage of $|\eta |< 1.48$ in the central barrel region and $1.48 <|\eta | < 3.00$ in the two forward endcap regions. The HCAL consists of a sampling calorimeter [28], which utilizes alternating layers of brass as an absorber and plastic scintillator as an active material, covering the range $|\eta |< 3$, and is extended to $|\eta |< 5$ by a forward hadron calorimeter. Muons are measured in the range $|\eta |< 2.4$ with detection planes which employ three technologies: drift tubes, cathode strip chambers, and resistive-plate chambers. The muon trigger combines the information from the three sub-detectors with a coverage up to $|\eta |<2.1$. A more detailed description of the CMS detector, together with a definition of the coordinate system used and the relevant kinematic variables, can be found in Ref. [28].

## Simulated samples

For the modelling of the background we use the MadGraph v5.1.3.30 [29] event generator to simulate the production of W boson and Drell–Yan events in association with jets, the powheg 1.0 r1380 [30–35] package to generate $\mathrm{t}\overline{\mathrm{t}}$ and single top quark events, and pythia v6.424 [36] for diboson (WW, WZ, and ZZ) processes. All simulated event samples are generated using the CTEQ6L1 [37] parton distribution functions (PDF) set, except for the powheg $\mathrm{t}\overline{\mathrm{t}}$ sample, for which the CT10 PDF set [38] is used. All the samples are then processed further by pythia, using the Z2* tune [39, 40] for simulation of parton showering and subsequent hadronization, and for simulation of the underlying event. The passage of the particles through the CMS detector is simulated using the Geant4 package [41]. All simulated background samples are normalized to the integrated luminosity of the recorded data, using inclusive cross sections determined at next-to-leading order, or next-to-next-to-leading order when available, calculated with mcfm v6.6 [42–45] and fewz v3.1 [46], except for the $\mathrm{t}\overline{\mathrm{t}}$ sample, for which Top++ v2.0 [47] is used.

To simulate the signature of interest, we use a model of a generic narrow spin-1 ${\mathrm{W}^{\prime }}$ resonance implemented with MadGraph. We verified that the kinematic distributions agree with those predicted by implementations of the LH, composite Higgs and HVT models in MadGraph. The resonance width differs in the three models, but in each case it is found to be negligible with respect to the experimental resolution. More details on the parameters used for interpretation of the models are given in Sect. 8.

Extra proton–proton interactions are combined with the generated events before detector simulation to match the observed distribution of the number of additional interactions per bunch crossing (pileup). The simulated samples are also corrected for observed differences between data and simulation in the efficiencies of the lepton trigger [16], the lepton identification/isolation [16], and the selection criteria identifying jets originating from hadronization of bottom quarks (b-tagged jets) [27].

## Reconstruction and selection of events

### Trigger and basic event selection

Candidate events are selected during data taking using single-lepton triggers, which require either one electron or one muon without isolation requirements. For electrons the minimum transverse momentum $p_{\mathrm {T}}$ measured at the high level trigger is 80$~\text {GeV}$, while for muons the $p_{\mathrm {T}}$ must be greater than 40$~\text {GeV}$.

After trigger selection, all events are required to have at least one primary-event vertex reconstructed within a 24$\text {\,cm}$ window along the beam axis, with a transverse distance from the nominal pp interaction region of less than 2$\text {\,cm}$  [48]. If more than one identified vertex passes these requirements, the primary-event vertex is chosen as the one with the highest sum of $p_{\mathrm {T}} ^{2}$ over its constituent tracks.

Individual particle candidates are reconstructed and identified using the CMS particle-flow (PF) algorithm [49, 50], by combining information from all subdetector systems. The reconstructed PF candidates are each assigned to one of the five candidate categories: electrons, muons, photons, charged hadrons, and neutral hadrons.

### Lepton reconstruction and selection

Electron candidates are reconstructed by clustering the energy deposits in the ECAL and then matching the clusters with reconstructed tracks [51]. In order to suppress the multijet background, electron candidates must pass quality criteria tuned for high-$p_{\mathrm {T}}$ objects and an isolation selection [52]. The total scalar sum of the $p_{\mathrm {T}}$ over all the tracks in a cone of radius $\varDelta R = \sqrt{{(\varDelta \eta )^2+(\varDelta \phi )^2} } = 0.3$ around the electron direction, excluding tracks within an inner cone of $\varDelta R = 0.04$ to remove the contribution from the electron itself, must be less than 5 $~\text {GeV}$. A calorimetric isolation parameter is calculated by summing the energies of reconstructed deposits in both the ECAL and HCAL, not associated with the electron itself, within a cone of radius $\varDelta R = 0.3$ around the electron. The veto threshold for this isolation parameter depends on the electron kinematic quantities and the average amount of additional energy coming from pileup interactions, calculated for each event. The electron candidates are required to have $p_{\mathrm {T}} > 90$ $~\text {GeV}$ and $|\eta | < 1.44$ or $1.57<|\eta |<2.5$, thus excluding the transition region between ECAL barrel and endcaps.

Muons are reconstructed with a global fit using both the tracker and muon systems [53]. An isolation requirement is applied in order to suppress the background from multijet events in which muons are produced in the semileptonic decay of B hadrons. A cone of radius $\varDelta R = 0.3$ is constructed around the muon direction. Muon isolation requires that the scalar $p_{\mathrm {T}}$ sum over all tracks originating from the interaction vertex within the cone, excluding the muon itself, is less than 10 % of the $p_{\mathrm {T}}$ of the muon. The muon candidates are required to have $p_{\mathrm {T}} > 50$ $~\text {GeV}$ and $|\eta | < 2.1$ in each selected event.

Events are required to contain exactly one lepton candidate (electron or muon). That is, events are rejected if they contain a second lepton candidate with $p_{\mathrm {T}} > 35$ $~\text {GeV}$ (electrons) or $p_{\mathrm {T}} > 20$ $~\text {GeV}$ (muons).

### Jets and missing transverse momentum reconstruction

Hadronic jets are identified by clustering PF candidates, using the FastJet v3.0.1 software package [54]. In the jet-clustering procedure, charged PF candidates associated with pileup vertices are excluded, to reduce contamination from pileup. In order to identify a Higgs boson decaying into bottom quarks, jets are clustered using the Cambridge–Aachen algorithm [55] with a distance parameter of 0.8 (“CA8 jets”). Only the highest $p_{\mathrm {T}}$ CA8 jet is used. Jets in the event are also identified using the anti-$k_{\mathrm {T}}$ jet-clustering algorithm [56] with a distance parameter of 0.5 (“AK5 jets”). AK5 jets are required to be separated from the CA8 jet by $\varDelta R > 0.8$. An event-by-event correction based on the projected area of the jet on the front face of the calorimeter is used to remove the extra energy deposited in jets by neutral particles coming from pileup. Furthermore, jet energy corrections are applied, based on measurements in dijet and photon+jet events in data [57]. Additional quality criteria are applied to the jets in order to remove spurious jet-like features originating from calorimeter noise [58]. The CA8 (AK5) jets are required to be separated from the selected electron or muon candidate by $\varDelta R>0.8$ (0.3). Only jets with $p_{\mathrm {T}} >30$ $~\text {GeV}$ and $|\eta |<2.4$ are allowed in the subsequent steps of the analysis. Furthermore, CA8 jets are not used in the analysis if their pseudorapidity falls in the region $1.0 <|\eta |< 1.8$, thus overlapping the barrel-endcap transition region of the silicon tracker. In that region, ’noise’ can arise when the tracking algorithm reconstructs many fake displaced tracks associated with the jet. The simulation does not sufficiently describe the full material budget of the tracking detector in that region, thus it does not accurately describe this effect. Without this requirement, a bias can be introduced in the b tagging, jet substructure and missing transverse momentum information, making this analysis systematically prone to that noise. The probability of signal events satisfying the requirement that the pseudorapidity of the CA8 jet falls outside the region $1.0 <|\eta |< 1.8$ is 80 % (92 %) for a resonance mass of 1.0 (2.5)$~\text {TeV}$.

A b tagging algorithm, known as the combined secondary vertex algorithm [27, 59], is applied to reconstructed AK5 jets to identify whether they originate from bottom quarks. This method allows the identification and rejection of the $\mathrm{t}\overline{\mathrm{t}}$ events as described in Sect. 4.6. The chosen algorithm working point provides a misidentification rate for light-parton jets of $\sim$1 % and an efficiency of $\sim$70 % [27]. The simulated events are reweighted event-by-event with the ratio of the b tagging efficiency in data and simulation, determined in a sample enriched with b-jets. The average value of the correction factor is 0.95. The same b tagging algorithm is also used to identify whether the CA8 jet comes from a Higgs boson decaying into bottom quarks, as described in Sect. 4.5.

The missing transverse momentum $p_{\mathrm {T}} ^\text {miss}$ is defined as the magnitude of the projection on the plane perpendicular to the beams of the negative vector sum of the momenta of all the reconstructed particles in an event. The raw $p_{\mathrm {T}} ^\text {miss}$ value is modified to account for corrections to the energy-momentum scale of all the reconstructed AK5 jets in the event. More details on the $p_{\mathrm {T}} ^\text {miss}$ performance in CMS can be found in Refs. [60, 61]. A requirement of $p_{\mathrm {T}} ^\text {miss} > 80\,(40)$ $~\text {GeV}$ is applied for the electron (muon) channel. The higher threshold for the electron channel is motivated by the higher contribution from the multijet background expected in the low-$p_{\mathrm {T}} ^\text {miss}$ range due to jets misidentified as electrons. The background is expected to be negligible in the muon channel, for which a lower $p_{\mathrm {T}} ^\text {miss}$ threshold can be used to preserve a higher efficiency for a low-mass signal.

### The $\mathrm {W}\rightarrow \ell \nu$ reconstruction and identification

The identified electron or muon is associated with the $\mathrm {W}\rightarrow \ell \nu$ candidate. The $p_{\mathrm {T}}$ of the undetected neutrino is assumed to be equal to the $p_{\mathrm {T}} ^\text {miss}$. The longitudinal component $p_{z,\nu }$ of the neutrino momentum is calculated following a method used originally for the reconstruction of the invariant mass of the top quark as described in Ref. [62]. The method aims to solve a quadratic equation that makes use of the known W boson mass. Kinematic ambiguities in the solution of the equation are resolved as in Ref. [62]. The four-momentum of the neutrino is used to build the four-momentum of the $\mathrm {W}\rightarrow \ell \nu$ candidate.

### The $\mathrm{H} \rightarrow \mathrm{b} \overline{\mathrm{b}}$ identification using jet substructure and b tagging

The CA8 jets are used to reconstruct the jet candidates from decays of Lorentz-boosted Higgs boson to bottom quarks. We exploit two techniques to discriminate against quark and gluon jets from the multijet background, including the requirement that the reconstructed jet mass be close to the Higgs boson mass, and b tagging methods that discriminate jets originating from the b quarks from those originating from lighter quarks or gluons.

First, we apply a jet-grooming technique [26, 63] to re-cluster the jet constituents, while applying additional requirements to remove possible contamination from soft QCD radiation or pileup. Different jet-grooming algorithms have been explored at CMS, and their performance on jets in multijet processes has been studied in detail [63]. In this analysis, we use the *jet pruning* algorithm [64, 65], which re-clusters each jet starting from all its original constituents using the CA algorithm iteratively, while discarding soft and large-angle recombinations at each step. The performance of the algorithm depends on the two parameters, $z_\text {cut}=0.1$ and $D_\text {cut}=m_{\text {jet}}/{p_{\text {T}}^{\text {jet}}}$, which define the maximum allowed hardness and the angle of the recombinations in the clustering algorithm, respectively. A jet is considered as an H-tagged jet candidate if its pruned mass, $m_{\text {jet}}$, computed from the sum of the four-momenta of the constituents surviving the pruning, falls in the range $110<m_{\text {jet}}<135$ $~\text {GeV}$. The $m_{\text {jet}}$ window is the result of an optimization based on signal sensitivity and on the constraints due to the higher bounds of the signal regions of other diboson analyses [25].

The simulation modelling of the pruned mass measurement for merged jets from heavy bosons has been checked using merged $\mathrm {W}\rightarrow \overline{\mathrm{q}}\mathrm {q}'$ decays in $\mathrm{t}\overline{\mathrm{t}}$ events with a $\ell$+jets topology [26]. The data are compared with $\mathrm{t}\overline{\mathrm{t}}$ events generated with MadGraph, interfaced to pythia for parton showering. The differences between recorded and simulated event samples in the pruned jet mass scale and resolution are found to be up to 1.7 and 11 %, respectively. In addition, the modelling of bottom quark fragmentation is checked through reconstruction of the top quark mass in these $\mathrm{t}\overline{\mathrm{t}}$ events [66].

To discriminate between quark and gluon jets, on one hand, and a Higgs-initiated jet, on the other, formed by the hadronization of two bottom quarks, we use a H tagging technique [27]. This procedure splits the candidate H-jet into two sub-jets by reversing the last step of the CA8 pruning recombination algorithm. Depending on the angular separation $\varDelta R$ of the two sub-jets, different b tagging discriminators are used to tag the H-jet candidate. If $\varDelta R>0.3$, then the b tagging algorithm is applied to both of the individual sub-jets of the CA8 jet; otherwise, it is applied to the whole CA8 jet. The chosen algorithm working point provides a misidentification rate of 10 % and an efficiency of 80 %. The ratio of the b tagging efficiency between data and simulation, in a sample enriched with b-jets from gluon splitting by requiring two muons within the CA8 jet, is used to reweight the simulated events.

### Final event selection and categorization

After reconstructing the W and Higgs bosons, we apply the final selections used for the search. Both the W and Higgs boson candidates must have a $p_{\mathrm {T}}$ greater than 200$~\text {GeV}$. In addition, we apply topological selection criteria, requiring that the W and Higgs bosons are approximately back-to-back, since they tend to be isotropically distributed for background events. In particular, the $\varDelta R$ distance between the lepton and the H-tagged jet must be greater than $\pi /2$, the azimuthal angular separation between the $p_{\mathrm {T}} ^\text {miss}$ and the H-tagged jet must be greater than 2.0 radians, and the azimuthal angular separation between the $\mathrm {W}\rightarrow \ell \nu$ and H-tagged jet candidates must be greater than 2.0 radians. To further reduce the level of the $\mathrm{t}\overline{\mathrm{t}}$ background, events with one or more reconstructed AK5 jets, not overlapping with the CA8 H-tagged jet candidate as described previously in Sect. 4.3, are analyzed. If one or more of the AK5 jets is b-tagged, the event is rejected. Furthermore, a leptonically decaying top quark candidate mass $m_\text {top}^\ell$ is reconstructed from the lepton, $p_{\mathrm {T}} ^\text {miss}$, and the closest AK5 jet to the lepton using the method described in Ref. [62]. A hadronically decaying top quark candidate mass $m_\text {top}^\mathrm {h}$ is reconstructed from the CA8 H-tagged jet candidate and the closest AK5 jet. Events with $120<m_\text {top}^\ell <240$ $~\text {GeV}$ or $160<m_\text {top}^\mathrm {h}<280$ $~\text {GeV}$ are rejected. The chosen windows around the top quark mass are the result of an optimization carried out in this analysis, taking into account the asymmetric tails at larger values due to combinatorial background. If several distinct WH resonance candidates are present in the same event, only the candidate with the highest-$p_{\mathrm {T}}$ H-tagged jet is kept for further analysis. The invariant mass of the WH resonance ($\text{ M }_{\mathrm {W} \mathrm{H} }$) is required to be at least 0.7$~\text {TeV}$. The signal efficiency for the full event selection ranges between $\sim$3 and $\sim$9 %, depending on the resonance mass.

## Modelling of background and signal

### Background estimation

After the full event selection, the two dominant remaining backgrounds are expected to come from W+jets and $\mathrm{t}\overline{\mathrm{t}}$ events. Backgrounds from $\mathrm{t}\overline{\mathrm{t}}$, single top quark, and diboson production are estimated using simulated samples after applying correction factors derived from control samples in data. For the W+jets background estimation, a procedure based on data has been developed to determine both the normalization and the $\text{ M }_{\mathrm {W} \mathrm{H} }$ shape.

**Fig. 2:**
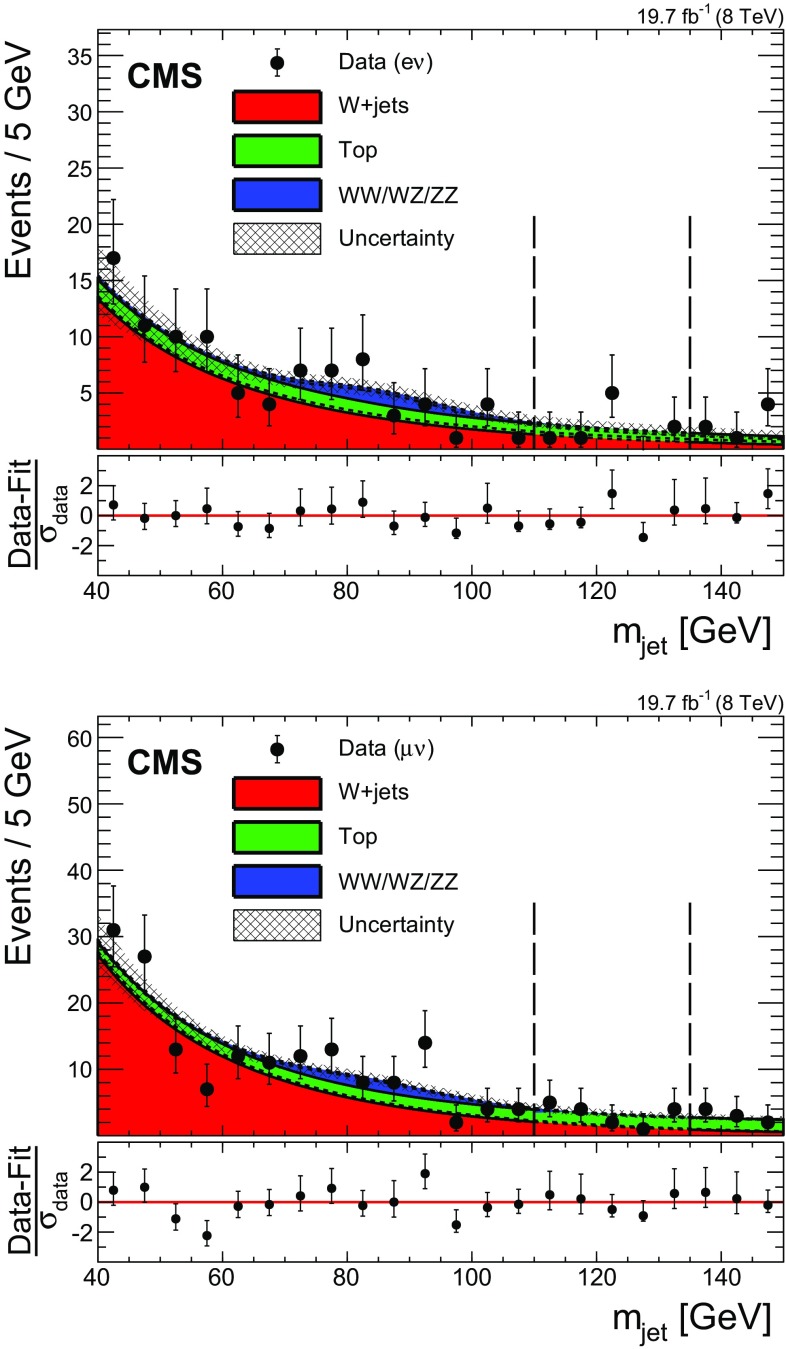
Distributions of the pruned jet mass, $m_{\text {jet}}$, in the electron (*top*) and muon (*bottom*) channels. The signal region lies between the *dashed vertical lines*. *The hatched region* indicates the statistical uncertainty of the fit. At the bottom of each plot, the bin-by-bin fit residuals, $(\text {Data}-\text {Fit})/\sigma _\text {data}$, are shown

For the W+jets normalization estimate, a signal-depleted control region is defined outside the $m_{\text {jet}}$ mass window described in Sect. 4.5. A lower sideband region is defined in the $m_{\text {jet}}$ range [40, 110]$~\text {GeV}$ as well as an upper sideband in the range [135, 150]$~\text {GeV}$. The overall normalization of the W+jets background in the signal region is determined from the likelihood of the sum of backgrounds fit to the $m_{\text {jet}}$ distribution in both sidebands of the observed data. In this approach, simulated events are replaced by an analytical function, which has been determined individually for each background process. Figure 2 shows the result of this fit procedure, where all selections are applied except the final $m_{\text {jet}}$ signal window requirement. The inclusive W+jets background is predicted from a fit excluding the signal region (between the vertical dashed lines), while the other backgrounds are estimated from simulation.

The shape of the W+jets background as a function of $\text{ M }_{\mathrm {W} \mathrm{H} }$ in the signal region is estimated using the lower sideband region of the $m_{\text {jet}}$ distribution. Correlations needed to extrapolate from the sideband to the signal region are determined from simulation through an extrapolation function defined as:

1 $$\begin{aligned} \alpha _\mathrm {MC}(\text{ M }_{\mathrm {W} \mathrm{H} }) = \frac{F_\mathrm {MC, SR}^{\mathrm {W}+\text {jets}}(\text{ M }_{\mathrm {W} \mathrm{H} })}{F_\mathrm {MC, SB}^{\mathrm {W}+\text {jets}}(\text{ M }_{\mathrm {W} \mathrm{H} })}, \end{aligned}$$

where $F_\mathrm {MC, SR}^{\mathrm {W}+\text {jets}}$ and $F_\mathrm {MC, SB}^{\mathrm {W}+\text {jets}}$ are the probability density functions determined from the $\text{ M }_{\mathrm {W} \mathrm{H} }$ spectrum in simulation for the signal region and low-$m_{\text {jet}}$ sideband region, respectively.

In order to estimate the W+jets contribution $F_{\text {DATA}, \mathrm {SB}}^{\text {W+jets}}$ in the control region of the data the other backgrounds are subtracted from the observed $\text{ M }_{\mathrm {W} \mathrm{H} }$ distribution in the lower sideband region. The shape of the W+jets background distribution in the signal region is obtained by scaling $F_{\text {DATA}, \mathrm {SB}}^{\mathrm {W}+\text {jets}}$ according to $\alpha _\mathrm {MC}$. The final prediction of the background contribution in the signal region, $N^\text {BKGD}_\mathrm {SR}$, is given by

2 $$\begin{aligned} N^\mathrm {BKGD}_\mathrm {SR}(\text{ M }_{\mathrm {W} \mathrm{H} })= & {} C_{\mathrm {SR}}^{\mathrm {W}+\text {jets}}\, F_{\text {DATA}, \mathrm {SB}}^{\mathrm {W}+\text {jets}}(\text{ M }_{\mathrm {W} \mathrm{H} })\, \alpha _\mathrm {MC}(\text{ M }_{\mathrm {W} \mathrm{H} })\nonumber \\&+ \sum _{k} C_{\mathrm {SR}}^{k}~F_\mathrm {MC, SR}^{k}(\text{ M }_{\mathrm {W} \mathrm{H} }), \end{aligned}$$

where the index *k* runs over the list of minor backgrounds, and $C_{\mathrm {SR}}^{\mathrm {W}+\text {jets}}$ and $C_{\mathrm {SR}}^{k}$ represent the normalizations of the yields of the dominant W+jets background and of the different minor background contributions. The $C_{\mathrm {SR}}^{\mathrm {W}+\text {jets}}$ parameter is determined from the fit to the $m_{\text {jet}}$ distribution as described above, while each $C_{\mathrm {SR}}^{k}$ is determined from simulation. The ratio $\alpha _\mathrm {MC}$ accounts for the small kinematic differences between signal and sideband regions, and is largely independent of the assumptions on the overall cross section. The validity and robustness of this method have been studied in data using a lower $m_{\text {jet}}$ sideband of [40, 80]$~\text {GeV}$ to predict an alternate signal region with $m_{\text {jet}}$ in the range [80, 110]$~\text {GeV}$. Both the normalization and shape of the W+jets background are successfully estimated for the alternate signal region. This alternate signal region differs from the signal region of the search for WW or WZ resonances in Ref. [25] as b tagging is applied to the CA8 jet. We are therefore able to evaluate the potential WW and WZ signal contamination in the alternate signal region and find less than 5 % signal contamination, assuming a signal cross section corresponding to the exclusion limit for a WW resonance from Ref. [25]. The $\text{ M }_{\mathrm {W} \mathrm{H} }$ distribution of the background in the signal and lower sideband regions is described analytically by a function defined as $f(x)\propto \exp [-x/(c_0+c_{1}x)]$, which is found to describe the simulation well. Alternative fit functions have been studied but in all cases the background shapes agree with that of the default function within uncertainties.

**Fig. 3:**
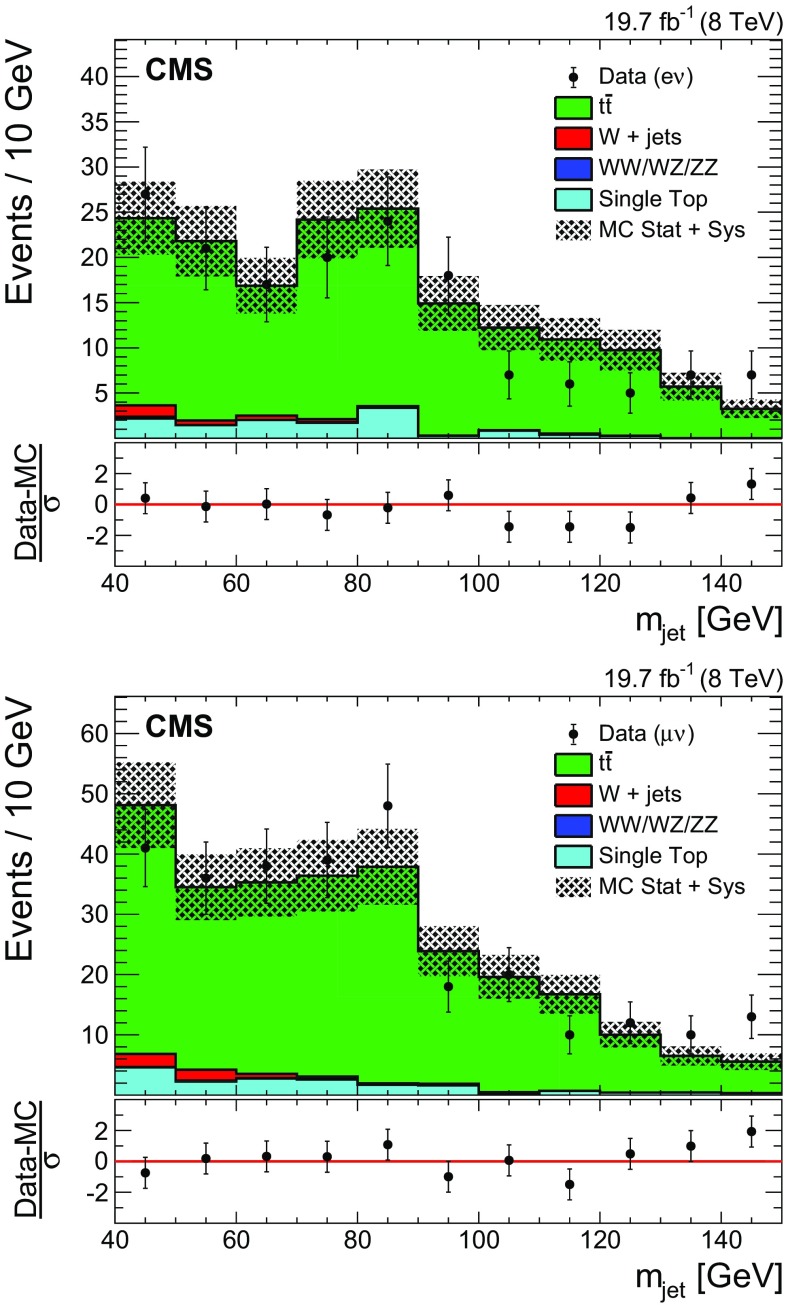
Distributions of $m_{\text {jet}}$ in the top quark enriched control sample in the electron (*top*) and muon (*bottom*) channels. *The hatched region* indicates the overall uncertainty in the background. In the lower panels, the bin-by-bin residuals, $(\text {Data}-\mathrm {MC})/\sigma$ are shown, where $\sigma$ is the sum in quadrature of the statistical uncertainty of the data, the simulation, and the systematic uncertainty in the $\mathrm{t}\overline{\mathrm{t}}$ background

For the $\mathrm{t}\overline{\mathrm{t}}$ background estimate, a control sample is selected by applying all analysis requirements, except that the b-tagged jet veto is inverted, the veto on the top quark mass is dropped, and the $m_{\text {jet}}$ requirement is removed. The data are compared with the predictions from simulation and good agreement is found. The pruned jet mass distribution in the top quark enriched control sample is shown in Fig. 3. The pruned jet mass distribution shows a small peak due to isolated W boson decays into hadrons, along with a smoothly varying combinatorial component mainly due to events in which the extra b-tagged jet from the top quark decay is in the proximity of the W boson. The difference in normalization between data and simulation is found to be $4.6 \pm 5.6$ %, where the quoted uncertainty is only statistical. This normalization difference is applied to correct the normalization of $\mathrm{t}\overline{\mathrm{t}}$ background in the signal region. The relative uncertainty of 5.6 % is used to quantify the uncertainty in the $\mathrm{t}\overline{\mathrm{t}}$ and single top quark background normalization, as described in Sect. 6.1.

### Modelling of the signal mass distribution

**Fig. 4:**
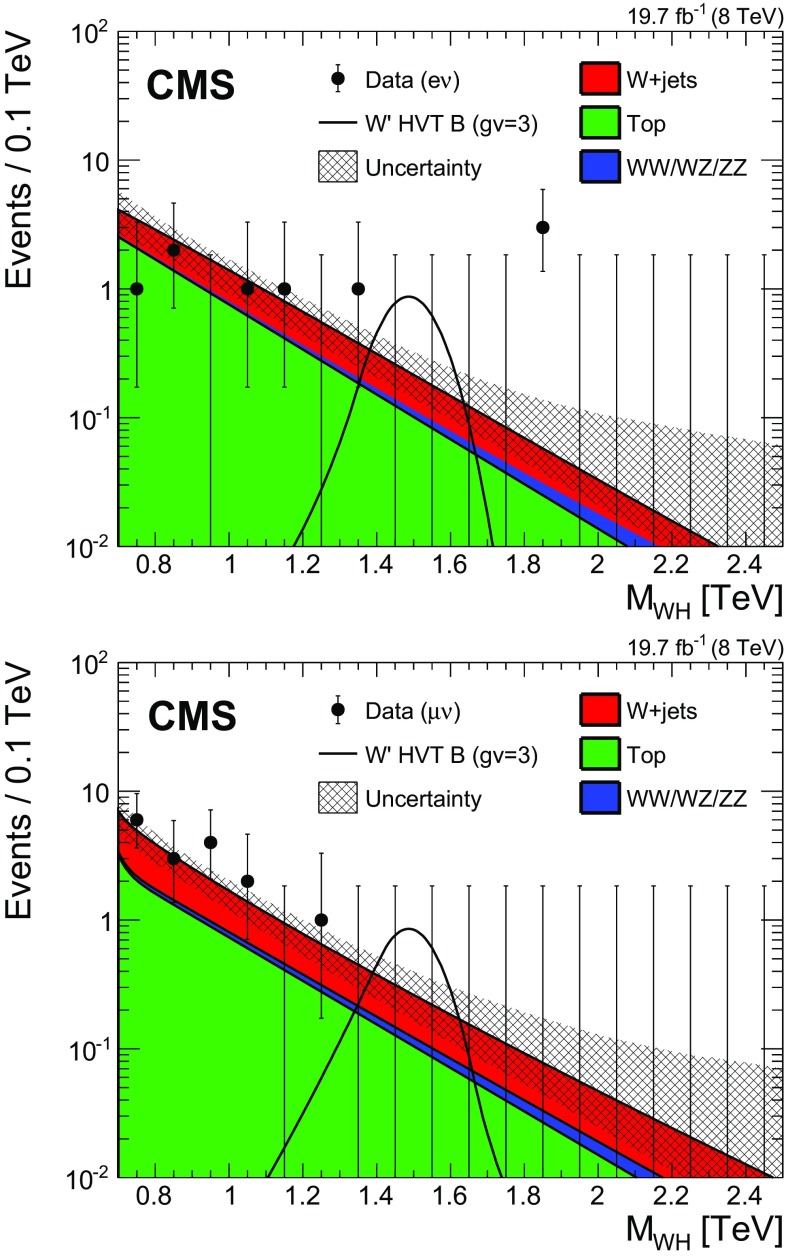
Final distributions in $\text{ M }_{\mathrm {W} \mathrm{H} }$ for data and expected backgrounds for electron (*top*) and muon (*bottom*) categories. The 68 % error bars for Poisson event counts are obtained from the Neyman construction [77]. *The hatched region* indicates the statistical uncertainty of the fit combined with the systematical uncertainty in the shape. This figure also shows a hypothetical ${\mathrm{W}^{\prime }}$ signal with mass of 1.5$~\text {TeV}$, normalized to the cross section predicted by the HVT model B with parameter $g_\mathrm {V} =3$ as described in Sect. 8.2

The shape of the reconstructed signal mass distribution is extracted from the simulated signal samples. In the final analysis of the $\text{ M }_{\mathrm {W} \mathrm{H} }$ spectrum, the statistical signal sensitivity depends on an accurate description of the signal shape. The signal shape is parametrized with a double-sided Crystal Ball function (i.e. a Gaussian core with power-law tails on both sides) [67] to describe the CMS detector resolution. Figure 4 shows an example of this parametrization for a ${\mathrm{W}^{\prime }}$ mass of 1.5$~\text {TeV}$. To take into account differences between the electron and muon $p_{\mathrm {T}}$ resolutions at high $p_{\mathrm {T}}$, the signal mass distribution is parametrized separately for events with electrons and muons. The resolution of the reconstructed $\text{ M }_{\mathrm {W} \mathrm{H} }$ is given by the width of the Gaussian core and is found to be 4–6 %.

## Systematic uncertainties

### Systematic uncertainties in the background estimation

Uncertainties in the estimation of the background affect both the normalization and the shape of the $\text{ M }_{\mathrm {W} \mathrm{H} }$ distribution. The systematic uncertainty in the W+jets background yield is dominated by the statistical uncertainty associated with the number of events in data in the $m_{\text {jet}}$ sideband regions, and it is found to be about 59 % (42 %) in the electron (muon) channel. The systematic uncertainty in the $\mathrm{t}\overline{\mathrm{t}}$  normalization comes from the data-to-simulation ratio derived in the top-quark-enriched control sample (5.6 %) as described in Sect. 5.1. The systematic uncertainties in the WW, $\mathrm {W}$ $\mathrm{Z}$, and ZZ inclusive cross sections are assigned to be 10 %, taken from the relative difference in the mean value between the CMS WW cross section measurement at $\sqrt{s}=8$ $~\text {TeV}$ and the SM expectation [68].

Systematic uncertainties in the W+jets background shape are estimated from the covariance matrix of the fit to the extrapolated data sideband and from the uncertainties in the modelling of $\alpha _\mathrm {MC}(\text{ M }_{\mathrm {W} \mathrm{H} })$. They are driven by the available data in the sidebands and the number of events generated for the simulation of the W+jets background, respectively. These uncertainties are shown in Fig. 4, and they are found to be about 30 % (120 %) at $\text{ M }_{\mathrm {W} \mathrm{H} } \approx 1~\text {TeV}$ (1.8$~\text {TeV}$). The estimation of the systematic uncertainty in the shape of the $\mathrm{t}\overline{\mathrm{t}}$ background takes into account the following contributions: the statistical uncertainty associated with the simulated event sample, the choices of regularization/factorization scales (varied up and down by a factor of 2), the matching scales in the MadGraph simulation, and an observed difference between MadGraph and powheg simulations.

Systematic effects from rare noise events identified in the tracker overlap region were specifically studied in the context of the acceptance requirement introduced for $\mathrm{H}$-jet candidates ($|\eta | < 1.0$ or $|\eta | > 1.8$) as described in Sect. 4. Those studies conclude that any residual noise effects following the imposition of this requirement are negligible. No additional source of systematic uncertainty is taken into account for the background predictions.

### Systematic uncertainties in the signal prediction

Systematic uncertainties in the signal prediction affect both the signal efficiency and the $\text{ M }_{\mathrm {W} \mathrm{H} }$ shape. The primary uncertainties in signal yields are summarized in Table 1 and described below.

**Table 1:** Summary of the systematic uncertainties in the signal yield, relative to the expected number of events

Source	Uncertainty [%]	
	Electron	Muon
Lepton trigger and ID efficiencies	3	2
Lepton $p_{\mathrm {T}}$ scale	$<$0.5	1
Lepton $p_{\mathrm {T}}$ resolution	$<$0.1	$<$0.1
Jet energy-momentum scale	1–3	
Jet energy-momentum resolution	$<$0.5	
Higgs boson mass tagging efficiency	2–10	
Higgs boson b tagging efficiency	2–8	
Unclustered energy scale	$<$0.5	
Pileup	0.5	
PDF	$<$0.5	
Integrated luminosity	2.6	

The systematic uncertainties in the signal efficiency due to the electron energy (E) and muon $p_{\mathrm {T}}$ scales are evaluated by varying the lepton E or $p_{\mathrm {T}}$ within one standard deviation of the corresponding uncertainty [51, 53]; the uncertainties due to the electron E and muon $p_{\mathrm {T}}$ resolutions are estimated applying a $p_{\mathrm {T}}$ and E smearing, respectively. In this process, variations in the lepton E or $p_{\mathrm {T}}$ are propagated consistently to the $p_{\mathrm {T}} ^\text {miss}$ vector. We also take into account the systematic uncertainties affecting the observed-to-simulated scale factors for the efficiencies of the lepton trigger, identification and isolation requirements. These efficiencies are derived using a specialized tag-and-probe analysis with $\mathrm{Z}\rightarrow \ell ^{+}\ell ^{-}$ events [69], and the uncertainty in the ratio of the efficiencies is taken as the systematic uncertainty. The uncertainties in the efficiencies of the electron (muon) trigger and the electron (muon) identification with isolation are 3 % (3 %) and 3 % (4 %), respectively.

The signal efficiency is also affected by the uncertainties in the jet energy-momentum scale and resolution. The jet energy-momentum scale and resolution are varied within their $p_{\mathrm {T}}$- and $\eta$-dependent uncertainties [57] to estimate their impact on the signal efficiency. The variations are also propagated consistently to the $p_{\mathrm {T}} ^\text {miss}$ vector.

The momentum scale uncertainty of particles that are not identified as leptons or clustered in jets (‘unclustered energy-momentum’) is found to introduce an uncertainty of less than 0.5 % in the signal efficiency.

We also include systematic uncertainties in the signal efficiency due to uncertainties in data-to-simulation scale factors for the pruned jet mass tagging, derived from the top quark enriched control sample [26] and b-tagged jet identification efficiencies [27]. These sources introduce a systematic uncertainty in the mass tagging and b tagging of the Higgs boson of 2–10 % and 2–8 %, respectively, depending on the signal mass.

The systematic uncertainty due to the modelling of pileup is estimated by reweighting the signal simulation samples such that the distribution of the number of interactions per bunch crossing is shifted according to the uncertainty in the inelastic proton–proton cross section [70, 71].

The impact of the proton PDF uncertainties on the signal efficiency is evaluated with the PDF4LHC prescription [72, 73], using the MSTW2008 [74] and NNPDF2.1 [75] PDF sets. The uncertainty in the integrated luminosity is 2.6 % [76].

In addition to systematic uncertainties in the signal efficiency discussed above, we consider uncertainties in the signal resonance peak position and width. The systematic effects that could change the signal shape are the uncertainties due to the $p_{\mathrm {T}}$/energy-momentum scale and resolution of electrons, muons, jets, and the unclustered energy-momentum scale. For each of these sources of experimental uncertainty, the energy-momentum of the lepton and jets, as well as the corresponding $p_{\mathrm {T}} ^\text {miss}$ vector, are varied (or smeared) by their relative uncertainties. The uncertainty in the peak position of the signal is estimated to be less than 1 %. The jet energy-momentum scale and resolution introduce a relative uncertainty of about 3 % in the signal width. The unclustered energy-momentum scale introduces an uncertainty in the signal width of 1 % at lower resonance masses ($<$1.5$~\text {TeV}$), and of 3 % at higher masses.

## Results

The predicted number of background events in the signal region after the inclusion of all backgrounds is summarized in Table 2 and compared with observations. The yields are quoted in the range $0.7 < \text{ M }_{\mathrm {W} \mathrm{H} } < 3$ $~\text {TeV}$. The expected background is derived with the sideband procedure. The uncertainties in the background prediction from data are statistical in nature, as they depend on the number of events in the sideband region. The muon channel has more expected background events than the electron channel owing to the lower $p_{\mathrm {T}} ^\text {miss}$ requirement on the muon and its worse mass resolution at high $p_{\mathrm {T}}$.

Figure 4 shows the $\text{ M }_{\mathrm {W} \mathrm{H} }$ spectra after all selection criteria have been applied. The highest mass event is in the electron category and has $\text{ M }_{\mathrm {W} \mathrm{H} } \approx 1.9$ $~\text {TeV}$. The observed data and the predicted background in the muon channel agree. In the electron channel, an excess of three events is observed with $\text{ M }_{\mathrm {W} \mathrm{H} } > 1.8$ $~\text {TeV}$, where about 0.3 events are expected, while in the muon channel no events with $\text{ M }_{\mathrm {W} \mathrm{H} } > 1.8$ $~\text {TeV}$ are observed, where about 0.3 events are expected.

**Table 2:** Observed and expected yields in the signal region together with statistical uncertainties

	$\mathrm {e}\nu$+H-jet	$\mu \nu$+H-jet
Observed yield	9	16
Expected total background	$11.3 \pm 3.1$	$14.9 \pm 3.1$
W+jets	$4.7 \pm 2.9$	$7.0 \pm 3.1$
Top	$6.3 \pm 1.1$	$7.3 \pm 0.4$
VV	$0.4 \pm 0.1$	$0.6 \pm 0.2$

## Statistical and model interpretation

### Significance of the data

A comparison between the $\text{ M }_{\mathrm {W} \mathrm{H} }$ distribution observed in data and the largely data-driven background prediction is used to test for the presence of a resonance decaying into WH. The statistical test is performed based on a profile likelihood discriminant that describes an unbinned shape analysis. Systematic uncertainties in the signal and background yields are treated as nuisance parameters and profiled in the statistical interpretation using log-normal priors.

**Fig. 5:**
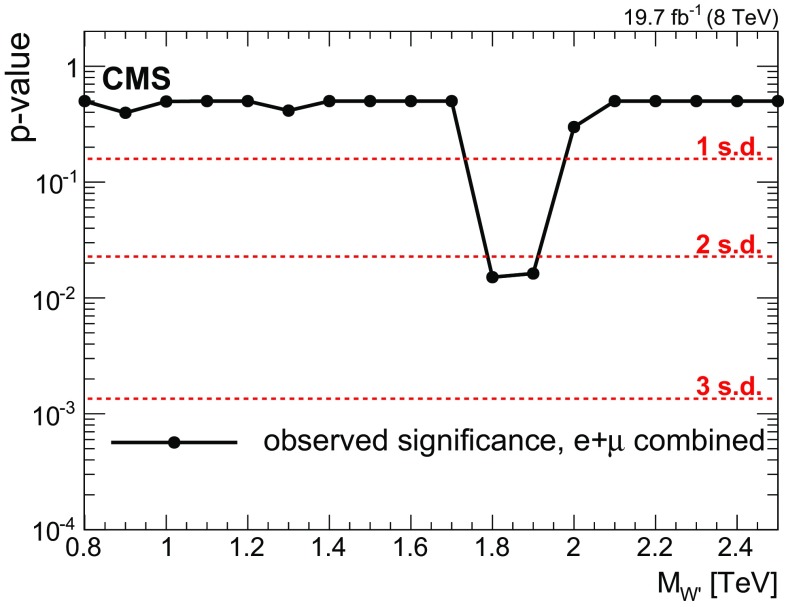
Local p-value of the combined electron and muon data as a function of the ${\mathrm{W}^{\prime }}$ boson mass, probing a narrow $\mathrm {W}\mathrm{H}$ resonance

**Table 3:** Intrinsic total widths ($\varGamma$) and cross sections ($\sigma$) for the LH model and HVT model B for different resonance masses. The $\mathrm {W}\mathrm{H} \rightarrow \ell \nu {\mathrm{b} \overline{\mathrm{b}} }$ branching fraction is not included in the calculation

Resonance mass [$\text {TeV}$ ]	LH model		HVT model B	
	$\varGamma$ [$\text {GeV}$ ]	$\sigma$ [pb]	$\varGamma$ [$\text {GeV}$ ]	$\sigma$ [pb]
0.8	7.22	$5.09\times 10^{-1}$	24.1	$3.37\times 10^{-1}$
0.9	8.12	$3.03\times 10^{-1}$	27.1	$2.48\times 10^{-1}$
1.0	9.02	$1.87\times 10^{-1}$	30.1	$1.71\times 10^{-1}$
1.1	9.92	$1.18\times 10^{-1}$	33.1	$1.16\times 10^{-1}$
1.2	10.8	$7.65\times 10^{-2}$	36.1	$8.05\times 10^{-2}$
1.3	11.7	$5.06\times 10^{-2}$	39.1	$5.59\times 10^{-2}$
1.4	12.6	$3.39\times 10^{-2}$	42.2	$3.88\times 10^{-2}$
1.5	13.5	$2.29\times 10^{-2}$	45.2	$2.51\times 10^{-2}$
1.6	14.4	$1.56\times 10^{-2}$	48.2	$1.87\times 10^{-2}$
1.7	15.3	$1.08\times 10^{-2}$	51.2	$1.30\times 10^{-2}$
1.8	16.2	$7.43\times 10^{-3}$	54.2	$9.03\times 10^{-3}$
1.9	17.1	$5.17\times 10^{-3}$	57.2	$6.27\times 10^{-3}$
2.0	18.0	$3.61\times 10^{-3}$	60.2	$4.25\times 10^{-3}$
2.1	19.0	$2.53\times 10^{-3}$	63.2	$3.02\times 10^{-3}$
2.2	19.8	$1.76\times 10^{-3}$	66.2	$2.10\times 10^{-3}$
2.3	20.8	$1.24\times 10^{-3}$	69.2	$1.46\times 10^{-3}$
2.4	21.6	$8.67\times 10^{-4}$	72.2	$1.01\times 10^{-3}$
2.5	22.6	$6.07\times 10^{-4}$	75.3	$7.31\times 10^{-4}$

We evaluate the local significance of the observations in the context of the described test, under the assumptions of a narrow resonance decaying into the WH final state and lepton universality for the W boson decay, by combining the two event categories. Correlations arising from the uncertainties common to both channels are taken into account. The result is shown in Fig. 5. The highest local significance of 2.2 standard deviations is found for a resonance mass of 1.8$~\text {TeV}$, driven by the excess in the electron channel described in Sect. 7. The corresponding local significance for a resonance of 1.8$~\text {TeV}$ in the electron channel is 2.9 standard deviations, while in the muon channel there is no significance. Taking into account the look-elsewhere effect [78], a local significance of 2.9 standard deviations translates into a global significance of about 1.9 standard deviations searching for resonances over the full mass range 0.8–2.5$~\text {TeV}$ and across two channels. We conclude that the results are thus statistically compatible with the SM expectation within 2 standard deviations.

### Cross section limits

We set upper limits on the production cross section of a new resonance following the modified-frequentist $CL_\mathrm {s}$ method [79, 80]. Exclusion limits can be set as a function of the ${\mathrm{W}^{\prime }}$ boson mass, under the narrow-width approximation. The results are interpreted in the HVT model B [15] which mimics the properties of composite Higgs scenarios, and in the context of the little Higgs model [8]. Typical parameter values for the HVT model B are

3 $$\begin{aligned} |c_\mathrm{H} | \approx |c_\mathrm {F} |\approx 1, \quad g_\mathrm {V} \ge 3, \end{aligned}$$

where $c_\mathrm{H}$ describes interactions involving the Higgs boson or longitudinally polarized SM vector bosons, $c_\mathrm {F}$ describes the direct interactions of the ${\mathrm{W}^{\prime }}$ with fermions, and $g_\mathrm {V}$ is the typical strength of the new interaction. In this scenario, decays of the ${\mathrm{W}^{\prime }}$ boson into a diboson are dominant and the ${\mathrm{W}^{\prime }}\rightarrow$ WH branching fraction is almost equal to that of the decay into WZ. The parameter points for this scenario are currently not well constrained from experiments [15] because of the suppressed fermionic couplings of the ${\mathrm{W}^{\prime }}$ boson.

The following parameters are used for interpretation of the results: $g_\mathrm {V} = 3$, $c_\mathrm{H} = -1$ and $c_\mathrm {F} = 1$ in the HVT model B and $\cot {2\theta } = 2.3$, $\cot {\theta } = -0.20799$ in the LH model, where $\theta$ is a mixing angle parameter that determines ${\mathrm{W}^{\prime }}$ couplings and that $\cot {2\theta }$ and $\cot {\theta }$ can be directly related to $c_\mathrm{H}$ and $c_\mathrm {F}$.

The intrinsic width and cross section for both models are listed in Table 3 for several resonance masses. The widths for the HVT model B are computed by means of Eqs. (2.25) and (2.31) in Ref. [15], while the cross sections were obtained using the online tools provided by the authors of Ref. [15]. The width is less than 5 % for the following parameter values: $0.95<g_\mathrm {V} <3.76$, $c_\mathrm{H} = -1$, and $c_\mathrm {F} = 1$; $g_\mathrm {V} <3.9$, $c_\mathrm{H} = -1$, and $c_\mathrm {F} = 0$; or $g_\mathrm {V} <7.8$, $c_\mathrm{H} = 0.5$, and $c_\mathrm {F} = 0$. The widths for the LH model have been computed by means of Eq. (15) in Ref. [81], and they are less than 5 % for values of $0.084<|\cot {\theta } |<1.21$. Hence, in both models we can consider the width to be negligible compared to the experimental resolution.

**Fig. 6:**
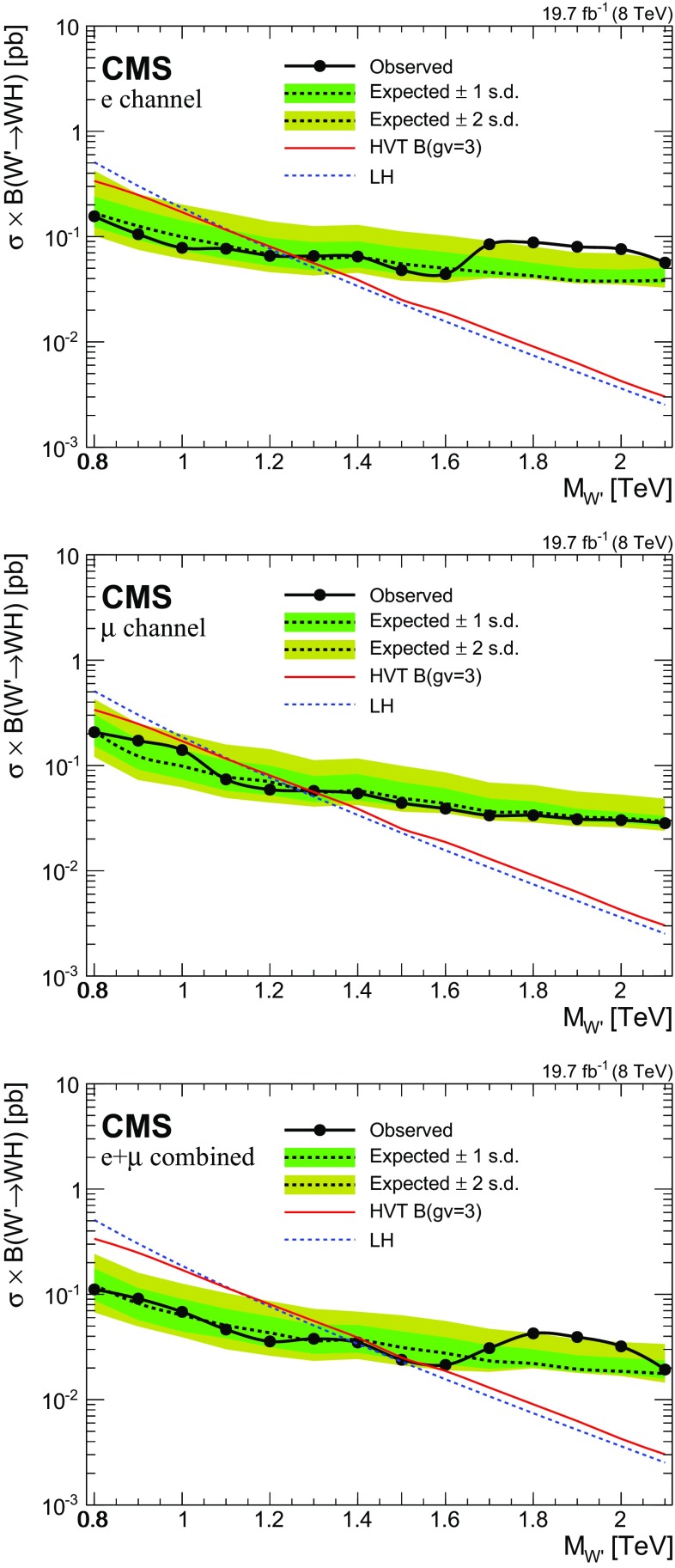
Observed (*solid*) and expected (*dashed*) upper limits at 95 % CL on the product of the ${\mathrm{W}^{\prime }}$ production cross section and the branching fraction of ${\mathrm{W}^{\prime }}\rightarrow \mathrm {W}\mathrm{H}$ for electron (*top*) and muon (*middle*) channels, and the combination of the two channels (*lower plot*). The products of cross sections and branching fractions for ${\mathrm{W}^{\prime }}$ production in the LH and HVT models are overlaid

Figure 6 shows the expected and observed exclusion limits at 95 % confidence level (CL) on the product of the ${\mathrm{W}^{\prime }}$ production cross section and the branching fraction of ${\mathrm{W}^{\prime }}\rightarrow \mathrm {W}\mathrm{H}$ for the electron and muon channels separately, and for the combination of the two. For the combined channels, the observed and expected lower limits on the ${\mathrm{W}^{\prime }}$ mass are 1.4$~\text {TeV}$ in the LH model and 1.5$~\text {TeV}$ in the HVT model B. For the electron (muon) channel, the observed and expected lower limits on the ${\mathrm{W}^{\prime }}$ mass are 1.2 (1.3)$~\text {TeV}$ in the LH model and 1.3 (1.3)$~\text {TeV}$ in the HVT model B.

### Analysis combination

**Fig. 7:**
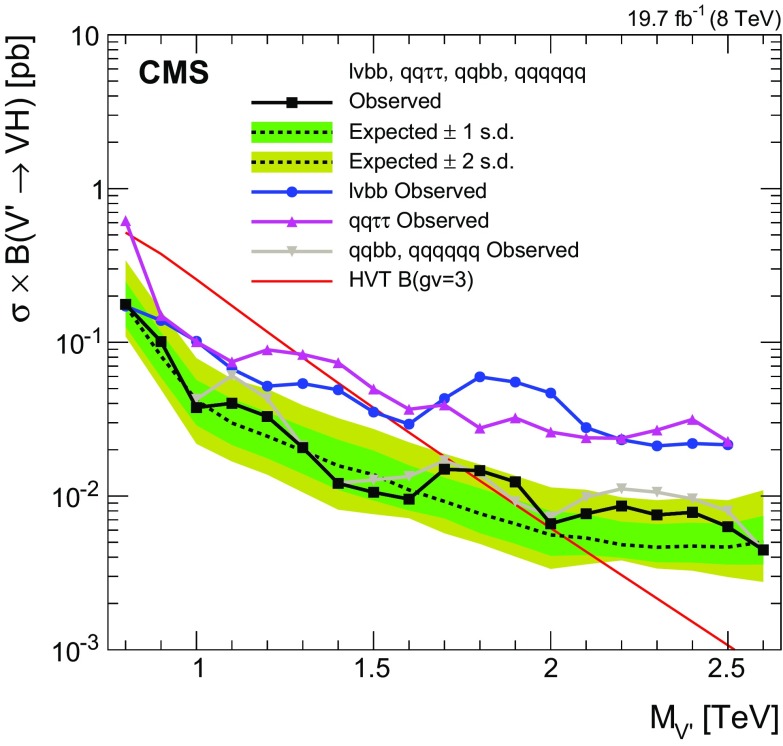
Observed (*full rectangles*) and expected (*dashed line*) combined upper limits at 95 % CL on the sum of the ${\mathrm{W}^{\prime }}$ and ${\mathrm{Z}}^\prime$ production cross sections, weighted by their respective branching fraction of ${\mathrm{W}^{\prime }}\rightarrow \mathrm {W}\mathrm{H}$ and ${\mathrm{Z}}^\prime \rightarrow \mathrm{Z}\mathrm{H}$. The cross section for the production of a ${\mathrm{W}^{\prime }}$ and ${\mathrm{Z}}^\prime$ in the HVT model B, multiplied by its branching fraction for the relevant process, is overlaid. The observed limits of the three analyses entering the combination in the final states, $\ell \nu \mathrm{b} \overline{\mathrm{b}}$ (*full circle*), ${\mathrm{q}} \overline{{\mathrm{q}}} \tau ^{+}\tau ^{-}$ [24] (*full triangle pointing up*), and ${\mathrm{q}} \overline{{\mathrm{q}}} \mathrm{b} \overline{\mathrm{b}} /{\mathrm{q}} \overline{{\mathrm{q}}} {\mathrm{q}} \overline{{\mathrm{q}}} {\mathrm{q}} \overline{{\mathrm{q}}}$ [22] (*full triangle pointing down*), are overlaid

The limits obtained in this analysis can be combined with two previous results [22, 24], setting limits on the sum of ${\mathrm{W}^{\prime }}\rightarrow \mathrm {W}\mathrm{H}$ and ${\mathrm{Z}}^\prime \rightarrow \mathrm{Z}\mathrm{H}$ production in the context of the HVT model. The search for ${\mathrm{W}^{\prime }}/\mathrm{Z}^{\prime } \rightarrow \mathrm {W}\mathrm{H}/{\mathrm{Z}} \mathrm{H} \rightarrow {\mathrm{q}} '\overline{{\mathrm{q}}} \mathrm{b} \overline{\mathrm{b}} /{\mathrm{q}} \overline{{\mathrm{q}}} {\mathrm{q}} \overline{{\mathrm{q}}} {\mathrm{q}} \overline{{\mathrm{q}}}$ [22] reports limits in the context of the HVT model that can be directly used in the combination. However, while an asymptotic approximation of the $CL_\mathrm {s}$ procedure was used in the original paper, for the combination the limit is re-evaluated with the $CL_\mathrm {s}$ procedure reported above. The search for $\mathrm{Z}^{\prime } \rightarrow {\mathrm{Z}} \mathrm{H} \rightarrow {\mathrm{q}} \overline{{\mathrm{q}}} \tau ^{+}\tau ^{-}$ [24], does not report limits in the context of a ${\mathrm{W}^{\prime }}$ resonance. However, since it is also sensitive to a signal from ${\mathrm{W}^{\prime }}\rightarrow \mathrm {W}\mathrm{H} \rightarrow {\mathrm{q}} '\overline{{\mathrm{q}}} \tau ^{+}\tau ^{-}$ with an efficiency of about 5 % less than for the $\mathrm{Z}^{\prime }$ signal, it was reinterpreted for the purpose of the combination. The results of the combination are shown in Fig. 7. The limit on the mass of the ${\mathrm{W}^{\prime }}/\mathrm{Z}^{\prime }$ is slightly improved to 1.8$~\text {TeV}$ compared to the most stringent result reported by the ${\mathrm{W}^{\prime }}/\mathrm{Z}^{\prime } \rightarrow \mathrm {W}\mathrm{H}/{\mathrm{Z}} \mathrm{H} \rightarrow {\mathrm{q}} ^\prime \overline{{\mathrm{q}}} \mathrm{b} \overline{\mathrm{b}} /{\mathrm{q}} \overline{{\mathrm{q}}} {\mathrm{q}} \overline{{\mathrm{q}}} {\mathrm{q}} \overline{{\mathrm{q}}}$ search.

**Fig. 8:**
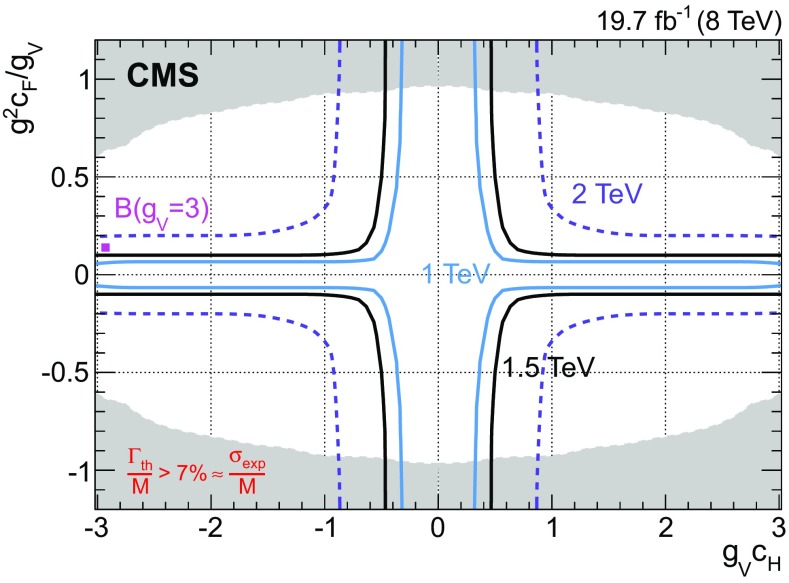
Exclusion regions in the plane of the HVT-model couplings ($g_\mathrm {V} c_\mathrm{H}$, $g^{2}c_\mathrm {F}/g_\mathrm {V}$) for three resonance masses, 1, 1.5, and 2$~\text {TeV}$, where *g* denotes the weak gauge coupling. The point B of the benchmark model used in the analysis is also shown. The boundaries of the regions outside these lines are excluded by this search are indicated by the *solid* and *dashed lines* (region outside these lines is excluded). The areas indicated by the *solid shading* correspond to regions where the resonance width is predicted to be more than 7 % of the resonance mass and the narrow-resonance assumption is not satisfied

In Fig. 8, a scan of the coupling parameters and the corresponding observed 95 % CL exclusion contours in the HVT model from the combination of the analyses are shown. The parameters are defined as $g_\mathrm {V} c_\mathrm{H}$ and $g^{2}c_{F}/g_\mathrm {V}$, related to the coupling strengths of the new resonance to the Higgs boson and to fermions. The range of the scan is limited by the assumption that the new resonance is narrow. A contour is overlaid, representing the region where the theoretical width is larger than the experimental resolution of the searches, and hence where the narrow-resonance assumption is not satisfied. This contour is defined by a predicted resonance width of 7 %, corresponding to the largest resonance mass resolution of the considered searches.

## Summary

A search has been presented for new resonances decaying into WH, in which the W boson decays into $\ell \nu$ with $\ell = \mathrm {e}$, $\mu$ and the Higgs boson decays to a pair of bottom quarks. Each event is reconstructed as a leptonic W boson candidate recoiling against a jet with mass compatible with the Higgs boson mass. A specialized b tagging method for Lorentz-boosted Higgs bosons is used to further reduce the background from multijet processes. No excess of events above the standard model prediction is observed in the muon channel, while an excess with a local significance of 2.9 standard deviations is observed in the electron channel near $\text{ M }_{\mathrm {W} \mathrm{H} } \approx 1.8~\text {TeV}$. The results are statistically compatible with the standard model within 2 standard deviations. In the context of the little Higgs and the heavy vector triplet models, upper limits at 95 % confidence level are set on the ${\mathrm{W}^{\prime }}$ production cross section in a range from 100 to 10$\text {\,fb}$ for masses between 0.8 and 2.5$~\text {TeV}$, respectively. Within the little Higgs model, a lower limit on the ${\mathrm{W}^{\prime }}$ mass of 1.4$~\text {TeV}$ has been set. A heavy vector triplet model that mimics the properties of composite Higgs models has been excluded up to a ${\mathrm{W}^{\prime }}$ mass of 1.5$~\text {TeV}$. In this latter context, the results have been combined with related searches, improving the lower limit up to $\approx$1.8$~\text {TeV}$. This combined limit is the most restrictive to date for ${\mathrm{W}^{\prime }}$ decays to a pair of standard model bosons.
